# Characterization and targeting of malignant stem cells in patients with advanced myelodysplastic syndromes

**DOI:** 10.1038/s41467-018-05984-x

**Published:** 2018-09-12

**Authors:** Brett M. Stevens, Nabilah Khan, Angelo D’Alessandro, Travis Nemkov, Amanda Winters, Courtney L. Jones, Wei Zhang, Daniel A. Pollyea, Craig T. Jordan

**Affiliations:** 1 Division of Hematology, Department of Medicine, University of Colorado School of Medicine, Aurora, CO 80045 USA; 2 Department of Biochemistry and Molecular Genetics, University of Colorado Denver, Aurora, CO 80045 USA; 3 Department of Pediatrics, University of Colorado Denver, Aurora, CO 80045 USA

## Abstract

Myelodysplastic syndrome (MDS) is a chronic hematologic disorder that frequently evolves to more aggressive stages and in some cases leads to acute myeloid leukemia (AML). MDS arises from mutations in hematopoietic stem cells (HSCs). Thus, to define optimal therapies, it is essential to understand molecular events driving HSC pathogenesis. In this study, we report that during evolution of MDS, malignant HSCs activate distinct cellular programs that render such cells susceptible to therapeutic intervention. Specifically, metabolic analyses of the MDS stem cell compartment show a profound activation of protein synthesis machinery and increased oxidative phosphorylation. Pharmacological targeting of protein synthesis and oxidative phosphorylation demonstrated potent and selective eradication of MDS stem cells in primary human patient specimens. Taken together, our findings indicate that MDS stem cells are reliant on specific metabolic events and that such properties can be targeted prior to the onset of clinically significant AML, during antecedent MDS.

## Abstract (web summary)

Myelodysplastic syndrome (MDS) arises from mutations in hematopoietic stem cells (HSCs). Here, the authors demonstrate that HSCs in higher-risk MDS express the surface marker CD123 and are characterized by activation of protein synthesis machinery and increased oxidative phosphorylation.

## Introduction

Myelodysplastic syndrome (MDS) is a malignant clonal hematopoietic disorder that leads to bone marrow failure in adults and can evolve to acute myeloid leukemia (AML). MDS is stratified into risk categories, defined by an IPSS (international prognostic scoring system) score, with low-risk associated with milder cytopenias, reduced progression to AML and longer survival, and high-risk associated with more severe cytopenias, greater progression to AML and shorter survival^1^. Patients with MDS who progress to AML have worse outcomes, with greater resistance to chemotherapy and higher treatment-related mortality rates^2,3^. Few advances have been made to change these poor outcomes, creating an immediate need for a deeper understanding of MDS and an urgency for novel therapies that can halt progression to AML. Central to developing improved therapies is a closer examination of the cells involved in the initiation/pathogenesis of MDS.

To better understand the pathogenesis of MDS, we drew upon the extensive literature available for AML. Multiple reports have shown a functionally defined subset of AML stem cells is able to recapitulate a human disease phenotype using in vitro and xenograft model systems^4–6^. We and others have extensively examined and further defined this subset on the basis of cell surface phenotype, gene expression, intracellular signaling, and metabolism^7–11^. AML stem cells have also been shown to be largely resistant to chemotherapy^12,13^. While MDS has long been hypothesized to be a stem cell disease, key events that define the pathogenesis of MDS stem cells are as yet unclear. Recently, one group has shown the existence of driver mutations in a subset of the hematopoietic stem cell (HSC) compartment defined by specific cell surface antigens^14^. These data suggest that key mutations occur at the HSC stage, thereby leading to the evolution of malignant stem cells and frank disease. Surface antigens have been used historically to define leukemic stem cells; our group and others have demonstrated that CD123 (IL3-R alpha chain) is a marker of AML stem cells that can be used to both delineate and target these cells^10,15,16^. Expression of CD123 in the CD34+/CD38− population of primary MDS cells has been reported^17^, and a subsequent study noted progressive increases of CD123 expression during MDS pathogenesis^18^. However, as yet, no molecular or functional analysis has been reported.

The findings in the present study indicate that upregulation of CD123 within the MDS stem cell compartment denotes a dramatic change in cellular physiology. The most significant change we observe is upregulation of protein synthesis machinery. Importantly, the correct homeostatic balance of protein synthesis in normal HSCs has been shown to play an integral role in self-renewal and survival^19^. Protein synthesis has also been shown to play a role in leukemogenesis. In the presence of Pten deletion, protein synthesis increased and subsequent aberrant leukemogenesis occurred^19^. Hence, altered intrinsic rates of protein synthesis suggest a profound change in the overall mechanisms regulating self-renewal of HSCs. Our data indicate that elevated protein synthesis represents a potentially attractive opportunity for therapeutic intervention and may be a means to selectively target MDS stem cells, while sparing normal stem cells. Further, our data suggest that CD123+MDS stem cells exhibit significant changes in cellular energy metabolism. We have previously shown in AML stem cells that energy metabolism, specifically oxidative phosphorylation, is directly linked to stem cell self-renewal and survival^7^. We and others have previously reported several strategies related to mitochondrial physiology and energy production that may be appropriate for targeting malignant stem cells in MDS^7,20,21^. These differences can be further exploited for a therapeutic benefit and as we show in the present study, the combination of targeting protein synthesis and energy metabolism has proven effective at eliminating MDS stem cells in in vitro culture and in a patient tissue derived MDS xenograft model. We propose that this type of approach can be readily translated to human therapeutic regimens, with the potential to eradicate MDS stem cells in patients with advanced stages of MDS.

## Results

### Phenotype of emergent malignant stem cells

**Fig. 1:**
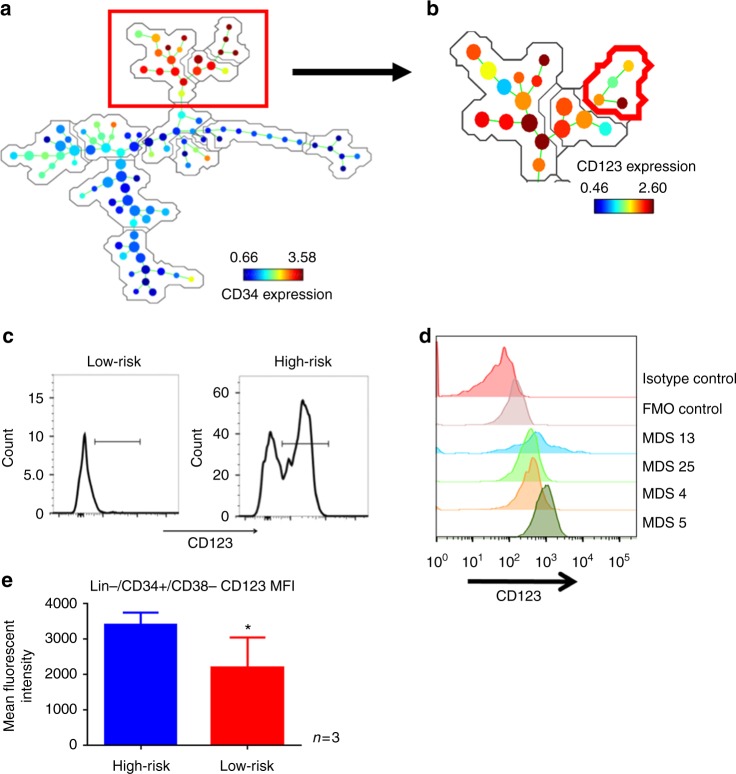
Investigation of CD123 expression in high-risk MDS. **a** Mass cytometry analysis of a representative high-risk MDS bone marrow specimen. The SPADE algorithm was used to cluster independent populations based on the expression of 14 independent cell surface markers. Blue to Red heat map coloring indicates relative expression of CD34 within each of the subpopulations identified by SPADE analysis. The red boxed cluster indicates all detected subpopulations defined as CD34+/Lin−. **b** Detailed analysis of CD34+ nodes. Indicated by red outline is the CD34+, CD38-, Lin- nodes. Heat map coloring indicates relative expression of CD123. **c**, **d** Flow cytometric analysis of CD34+/CD38−/Lin− MDS bone marrow cells labeled with CD123. Panel **c** shows comparison of representative low vs. high-risk specimens. Panel **d** shows additional high-risk MDS specimens in comparison to an isotype control. **e** Aggregate data of CD123 mean fluorescent intensity in high-risk vs. low-risk MDS patient samples showing a significant increase in high-risk MDS. See supplementary figure 1A for analysis of additional specimens. * *p* < 0.05 (two-tail *t*-test) error bars are S.D

To better understand the immunophenotype of MDS stem cells, we employed a high-dimensional analysis of cell surface antigens utilizing mass cytometry and flow cytometry. The unbiased analysis and clustering of mass cytometry data confirmed previous reports^17,18^ of a CD123 positive population in the primitive compartment of high-risk MDS patient bone marrow specimens. An example of this analysis (Fig. 1a, b and supplementary figure 1-2) shows a high-dimensional representation of CD34 expression within a typical MDS specimen. The data, which represent use of 14 different cell surface markers to identify various phenotypes (clustering markers noted in supplementary table 4), illustrate the numerous and very heterogeneous subpopulations in which CD34 is expressed (expression level indicated by color bar). The red boxed area in Fig. 1a indicates the CD34+/Lin− phenotype, which typically contains the stem cell population. Figure 1b shows that CD123 is expressed within the stem cell compartment, but levels vary substantially, indicating that stem cells can be further subdivided into multiple phenotypically distinct subpopulations as shown by the presence of several unique nodes. We also see overlap in the CD123+ population in the primitive compartment with traditional HSC markers such as CD90 (supplementary fig. 2a). The SPADE analysis (colored for CD123 expression) shows that nodes labeled 123+ fit into a traditional HSC definition of Lin−/CD34+/CD38−/CD90+. While there are other nodes exhibiting CD123 positivity, these can be defined as more CMP or GMP like cells and are labeled as such in the plot. Further analysis of MDS patient specimens using conventional flow cytometry corroborated the presence of a CD123+ subpopulation within the primitive compartment (Lin−/CD34+/CD38−) of high-risk specimens, but not in low risk MDS patients (Fig. 1c–e and supplementary fig. 1) with significant differences found in the mean fluorescent intensity between low-risk and high-risk patients (Fig. 1e)^18,22^. Lastly, we note that CD123 expression is variable in the high-risk patients (Fig. 1d) as previously found in both MDS and AML^10,16,18^. As shown in supplementary fig. 1C, the frequency of the CD123+ subpopulation is variable amongst patients across the total marrow, frequency of blasts, and frequency in the CD34+/CD38− primitive cells. However, these frequencies are similar to what has been previously reported^18^. Interestingly, expression of the only other known MDS stem cell marker, CD99^23^, shows an increased mean fluorescence in CD34+/CD38−/CD123+ cells (Supplementary fig. 1B). Together, these findings indicate that CD123 is upregulated in the stem cell compartment during progressive stages of MDS evolution.

**Fig. 2:**
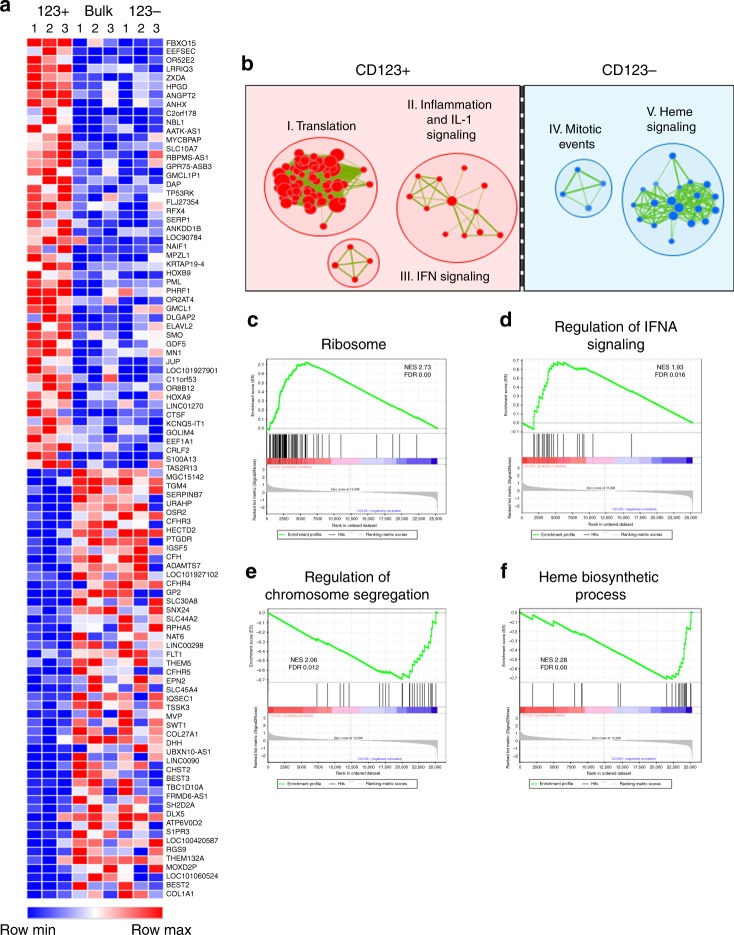
Whole-transcriptome analysis shows an upregulation of ribosome signaling in CD123+ MDS cells. RNA-seq was performed on Lin−/CD34+/CD38−/CD123+ or Lin−/CD34+/CD38−/CD123− primary MDS BM cells from three independent patients. **a** Heat map shows the top 50 up and downregulated genes. **b** Using Enrichment map algorithm, top pathways and groups of pathways were delineated in CD123+ vs. CD123− cells. **c**–**f** Using the KEGG database, the ribosome signature was the most significantly enriched gene set in the translation cluster of nodes in CD123+ cells (panel **b**). **d** The Regulation of IFNA Signaling gene set was also highly enriched in IL1 signaling group of nodes in CD123+ cells. **e** The Regulation of Chromosome Segregation was highly enriched in the mitotic events cluster of nodes in CD123- cells. **f** The Heme Biosynthetic Process pathway was highly enriched in the Heme signaling pathway in CD123- cells

**Fig. 3:**
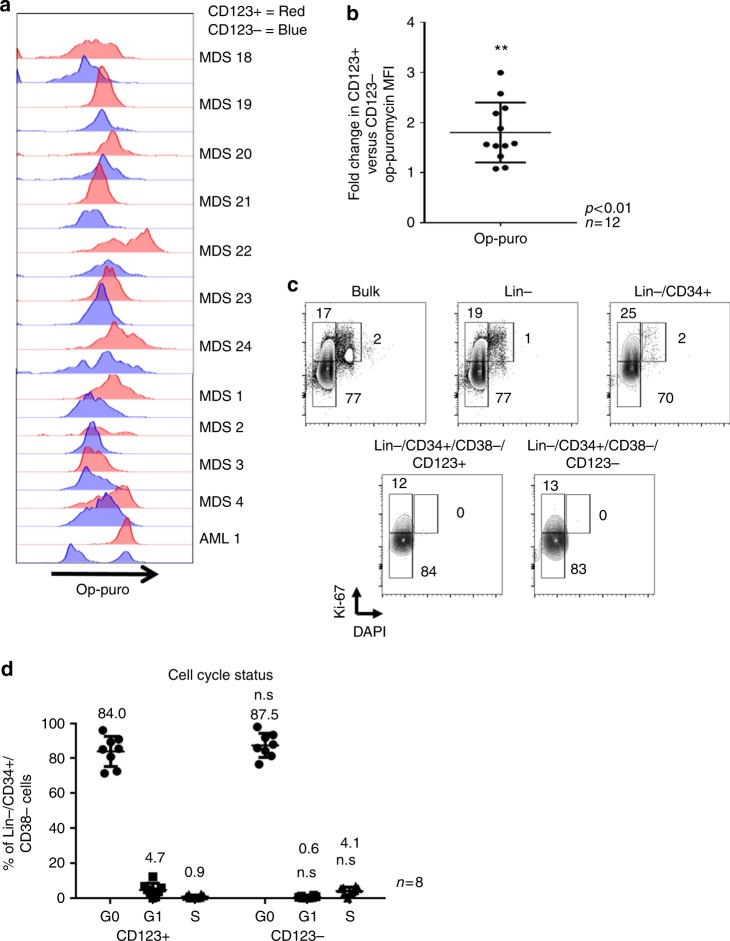
CD123+ stem cells exhibit increased translation but no change in cell cycle status. **a** CD123+ cells(red histogram) vs. CD123- cells(blue histogram) from Lin−/CD34+/CD38− high-risk bone marrow specimens exhibit increased protein synthesis as measured by op-puromycin in 11 independent patient specimens **b** Ratio of the mean fluorescence of CD123+ vs. CD123- cells shows an average 2-fold increase in CD123+ cells. **c** Cell cycle analysis of a representative MDS specimen (MDS 4) showing bulk, lin−, lin−/CD34+ and CD123+/CD123− stem cell populations. **d** Aggregate data comparing cell cycle in eight separate patient specimens shows no significant differences in CD123+ vs. CD123− cells. **p < .05 (two-tail *t*-test) error bars are S.D

To further investigate the role of the CD123+ subpopulation in the progression of MDS, sorted CD123+ cells from the Lin−/CD34+/CD38− compartment (hereafter termed “stem cells”) of three high-risk MDS specimens were isolated and subjected to whole-transcriptomic analysis. For comparison, CD123− cells in the primitive compartment and whole bone marrow mononuclear cells were also evaluated (Fig. 2a). Among the most strongly upregulated individual genes in the CD123+ population, were *HOXA9*^24^, *MN1*^25^, and *SMO*^26^. *HOXA9* has been studied extensively in AML and has been shown to be overexpressed in CD34+ cells of MDS patients^24^ and is known to play a role in leukemogenesis and transformation^27^. *MN1* has also been shown to play a role in AML and has been correlated to poor response and decreased survival. It has a role in the cell of origin of AML through the control of histone methyltransferases and creating a block in differentiation and inducing proliferation^28^. *SMO* has been shown to play a role in sensitivity to 5-azacytidine in MDS. An RNAi screen showed knockdown of *SMO* in combination with 5-azacytidine led to increased cytotoxicity^26^. To better understand global and pathway differences between CD123+ and CD123− cells we also performed gene set enrichment analysis (GSEA)^29^. These results were compiled into the enrichment map shown in Fig. 2b, supplementary fig. 4, and supplementary table 1^30^. This algorithm clusters gene sets to show functional groups as a collection of nodes. These nodes are grouped and annotated by their major phenotypic characteristics. In comparing CD123+ (red groups) and CD123− (blue groups) there are clear differences in multiple functions. In CD123+ cells there are increases in translation, IL-1 signaling and inflammation, and interferon signaling in contrast to changes in mitotic signaling and heme biosynthesis in CD123- cells. Previous groups have reported differences in IL-1 signaling in HSCs, MDS, and AML LSCs^31–33^. As shown previously by Barreyo et al. we find differences in *IL1RAP* levels in MDS specimens with chromosome 7 alterations; however, the differences in IL1 signaling is not due to *IL1RAP* expression alone as there are no differences between CD123+ and CD123- subpopulations (supplementary fig. 4D)^31^. Differences in heme biosynthesis have also recently been reported in MDS and play a role in the anemia found in most patients^34^. We further examined these clusters of nodes in CD123+ cells (Fig. 2c, d) and CD123− cells (Fig. 2e, f). This analysis showed the KEGG Ribosome pathway as the most strongly upregulated gene set in CD123+ cells vs. CD123− stem cells (Supplementary Table 1, Fig. 2c). The ribosomal subunits are almost globally overexpressed in the CD123+ cells. Many of these subunits contribute to the assembly of the large and small ribosomal subunits and protein production^35,36^. Wu et al. showed *RPL23*, a ribosomal gene was overexpressed in MDS CD34+ cells and associated with poor drug response^37^. Belickova et al. also showed increased expression of multiple ribosomal genes correlates with poor azacitidine response and disease progression^38^. We further confirmed one of the top hits, *RPL5*, showed significantly increased expression in the CD123+ compartment of the primitive compartment of a high-risk MDS patient in comparison to the CD123− compartment and the primitive compartment (Lin−/CD34+/CD38−) of normal bone marrow (Supplementary fig. 3A-B). These results demonstrate differential expression of ribosomal genes in primitive CD123+MDS cells and indicate a fundamental change in the physiology of malignant stem cells concomitant with expression of CD123. Additional gene sets found in the enrichment analysis represent known AML LSC targets including the JAK-STAT pathway^39^ and interestingly the spliceosome pathway (Supplementary Table 1). The upregulation of STAT3 signaling was found previously by Will et al. We found a similar upregulation of STAT3 signaling although not directly related to increased STAT3 levels^40^. We see gene-set enrichment and a small but significant increase in STAT3 Serine 727 phosphorylation (supplementary fig. 4). Crews et al. recently showed that the same spliceosome gene set is enriched in secondary AML in LSCs and contributes to disease progression^41^. We also see an enrichment for the REACTOME_ACUTE_MYELOID LEUKMIA gene set in the CD123+ subpopulation (Supplementary table 1). Thus, collectively, the gene expression changes observed in the CD123+ subpopulation are all consistent with transformation to AML. The upregulation of translation gene sets indicates a potential for increased protein synthesis in the CD123+ stem cell population. To further investigate these changes in translational activity we utilized a method previously described by Signer et al. to characterize the protein synthesis levels in HSCs^19^. The approach involves culturing cells with a fluorescent substrate known as OP-puro, which is incorporated into newly synthesized polypeptide chains and can be quantified as a measure of overall protein synthesis activity. Using this method, our results show that CD123+ stem cells exhibit markedly higher levels of protein translation in 12 separate patient samples (Fig. 3a). While the overall translation rates are variable there is a significant increase in translation rates between Lin−/CD34+/CD38−/CD123+ and Lin−/CD34+/CD38−/CD123− cells (Fig. 3b). We also see increased translation in the CD123+ subpopulation over lymphocytes and similar rates to monocytes in MDS patient specimens (supplementary fig. 9B). This degree of change could be due to increased proliferation or related activity; however, cell cycle analyses clearly demonstrate virtually identical profiles for the two populations, with both largely in the G0 phase of the cell cycle (84% in CD123+ vs. 88% in CD123−) for eight independent patient specimens (Fig. 3c, d and supplementary fig. 5a). Utilizing EdU staining we find similar rates of G0 as found with Ki67 and no differential between CD123+ and CD123− subpopulations (supplementary fig. 5b). Thus, as previously reported for AML, the malignant stem cell population retains a mostly quiescent phenotype, similar to normal HSCs^42^.

### Metabolic characteristics of CD123+ MDS stem cells

**Fig. 4:**
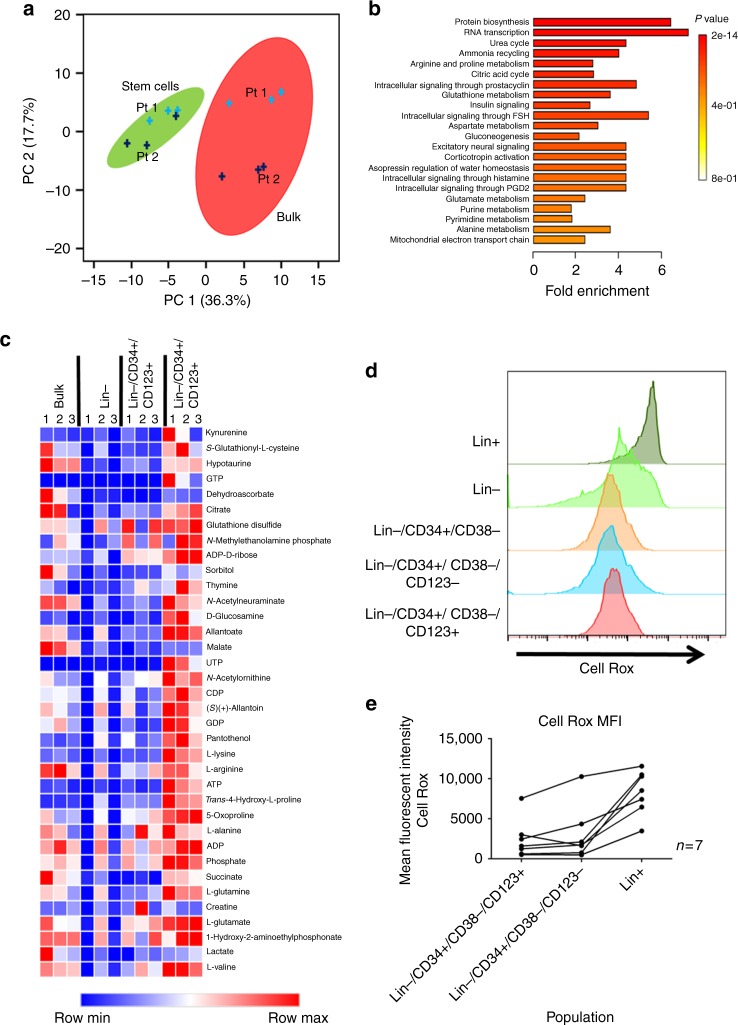
CD123 positive cells have a distinct metabolic phenotype. HPLC-based metabolomic analysis was done on sorted Lin−/CD34+/CD123+ cells from two high-risk MDS patients. **a** Principle component analysis of all measured metabolites shows similarities between CD123+ MDS stem cells from both patients (green circle). Bulk lineage negative cells (red circle) show distinctly different profiles. **b** Pathways most enriched in CD123+ MDS cells compared to CD123- population as determined by Metaboanalyst software analysis of metabolomics data. **c** Heat map representation of major metabolite differences including ATP, Glutamate, and TCA cycle intermediates. Specimen analyzed in triplicate (derived from patient 4—Supplementary Table 3). **d** Oxidative state analysis as indicated by CellROX dye labeling. **e** Aggregate data of CellROX labeling shows no significant difference between CD123+ vs. CD123− cells

To further analyze the biological properties of MDS stem cells, we performed studies to characterize cellular metabolism. This line of investigation was prompted by previous studies in which the metabolic state of AML stem cells has been shown to be distinct from bulk tumor and highly relevant to potential therapeutic intervention^7^. Indeed, energy metabolism plays an important role in the biology of malignant stem cells^7,43,44^. For these studies we sorted CD34+/CD123+, CD34+/CD123−, and lineage- cells from primary high-risk MDS specimens and performed comprehensive LC-MS metabolomic analyses^45^. We have recently developed improved methods that permit high-resolution metabolomic analyses with as few as 50,000 cells^46^. As shown in Fig. 4a, and consistent with studies of leukemia^47,48^, the malignant stem cell compartment is biologically distinct from bulk cells. These data indicate a fundamentally different physiology for the stem cell population. In comparing the CD123+ vs. CD123− stem cell subpopulations, we see strong upregulation of protein synthesis and RNA transcription (Fig. 4b and supplementary fig. 6), further confirming our findings from the gene expression and translational studies shown in Figs. 2 and 3a. The enrichment of protein synthesis and RNA transcription in CD123+ populations is also evidenced by increases in the majority of amino acids detected by mass spectrometry analysis (Supplementary fig. 6C). Energy metabolism was also distinct in the CD123+ MDS cells. The third, fifth, and sixth most enriched pathways are related to oxidative phosphorylation and the citric acid cycle (Fig. 4b and supplementary fig. 6). Mitochondrial pathways of this type are known to be deregulated in AML LSCs^7,20,21^ and their upregulation in high-risk MDS stem cells likely represents a key step in the transformation from chronic disease to AML. Figure 4c shows the individual metabolites contributing to these pathways that are increased in the CD123+ subpopulation. These metabolites include multiple components of the TCA cycle (e.g., l-glutamate, citrate, l-glutamine, succinate, malate). In addition, we detect increased components of glutathione metabolism (e.g., S-glutathionyl-L cysteine and glutathione disulfide), another characteristic of AML stem cells. Indeed, despite changes that occur during malignant transformation, we and others have reported that cancer stem cells retain a ROS (reactive oxygen species) low phenotype, a hallmark of normal HSCs^7^. Notably, our analysis of MDS specimens (Fig. 4d, e) shows a ROS-low phenotype for primitive cells in 7 patient specimens (Lin−/CD34+/CD38−/CD123+). Thus, we propose that increased glutathione metabolism as stem cells transition to more acute stages of malignancy may be important for maintaining a ROS-low status and therefore conditions conducive to self-renewal or related stem cell functions.

### Targeting unique metabolic properties of MDS stem cells

**Fig. 5:**
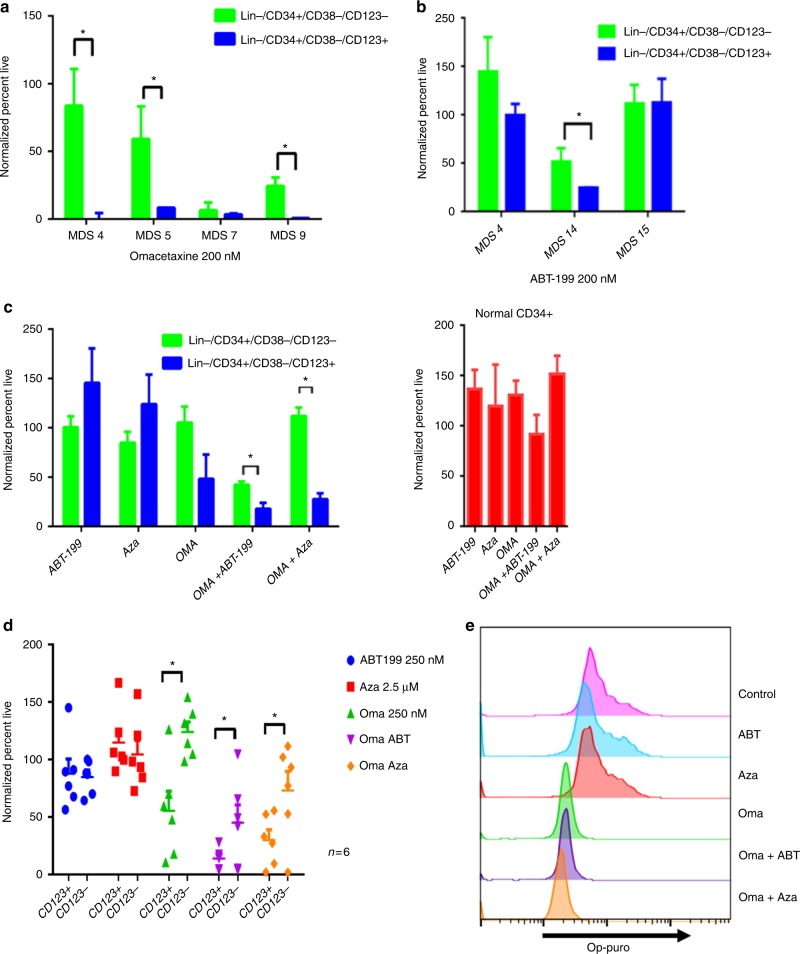
CD123 positive cells show differential sensitivity to multiple agents. Viability of primary MDS specimens following overnight culture in the presence of 200 nM omacetaxine, a protein synthesis inhibitor (**a**), or 200 nM ABT-199, a Bcl2 inhibitor (**b**). Data gated on CD123- vs. CD123+ primitive populations (Lin−/CD34+/CD38−). **c** Viability of a typical MDS specimen following overnight culture in varying drug conditions (left panel) in comparison to normal bone marrow CD34+ cells (right panel). ABT-199 (200 nm), OMA (omacetaxine, 200 nM), and Aza (azacitidine, 2.5uM). * indicates *p* < 0.01; bars represent mean ± S.D. from three replicates. **d** Six independent patient samples showing toxicity to drugs used in panel **c** comparing CD123+ vs. CD123- cells. **e** Protein synthesis levels following 4 h of treatment with omacetaxine,ABT-199, azacitidine, omacetaxine plus ABT-199, or omacetaxine plus azacitidine as indicated by OP-puromycin labeling. *p < .05 (two-tail *t*-test) error bars are S.D

As a strategy towards improving therapeutic outcomes for MDS, we investigated targeting of pathways deregulated in the CD123+ MDS stem cell compartment. Based on the findings outlined in Figs. 2, 3, 4, we first tested strategies that involve inhibiting protein translation. Initial studies employed a reagent grade compound, anisomycin, known to inhibit protein translation and induce so-called “ribotoxic stress”^49^. This agent showed selective killing of CD34+/CD38−/CD123+ cells at 200 nM and 400 nM (Supplementary fig. 8), suggesting that primary MDS cells with elevated protein synthesis were indeed susceptible to drugs of this class. Subsequent studies employed the drug omacetaxine mepesuccinate, a protein synthesis inhibitor which is FDA approved for the treatment of chronic myeloid leukemia^50^, and has also shown activity in the treatment of AML^51^. Notably, omacetaxine specifically targets CD123+ cells in multiple patient samples and is effective at decreasing protein translation levels (Figs. 5a, c–e). Omacetaxine is also preferentially active against the CD123+ stem cell compartment, as shown in Fig. 5a. Due to its labeled indication for the treatment of MDS, we also tested the effects of azacitidine (Fig. 5c, d). In most cases, we observed little activity for this agent towards the MDS stem cell population with no significant decreases in viability in CD123+ cells and no differential toxicity between CD123+ vs. CD123− in seven high-risk MDS patient specimens.

The second major phenotype found to be deregulated in CD123+ cells was oxidative phosphorylation and redox status (Fig. 4 and supplementary fig. 6). We have previously reported that BCL2 inhibition acts to suppress oxidative phosphorylation in primary AML cells. Thus, we investigated the BCL-2 inhibitor ABT-199 (venetoclax) for treatment of MDS stem cells. As shown in Fig. 5b, ABT-199 alone shows preferential toxicity to CD123+ cells, albeit with varying degrees of efficacy. The single drug resistance to ABT-199 found in certain patients is overcome with the combination of a protein translation inhibitor (anisomycin or omacetaxine) (Fig. 5c and supplementary fig. 8B). Thus, the combination of inhibiting protein synthesis and BCL2-mediated events showed enhanced and selective targeting of CD123+ MDS stem cells. Notably, analysis of the underlying metabolic consequences of the combined drug treatment supports the role of oxidative phosphorylation in survival of MDS cells. As shown in supplementary fig. 5, treatment of primary MDS cells with either ABT-199 or omacetaxine alone impairs mitochondrial respiration. However, the combination of the two is markedly superior, with clear inhibition of basal oxygen consumption, maximal respiration, and ATP production (supplementary fig. 7). An additional component of the efficacy observed with combined ABT-199 and omacetaxine may involve suppression of MCL-1. As shown in supplementary fig. 2, we observe increased MCL-1 expression in CD34+/CD123+ MDS cells. Resistance to BCL-2 inhibition can be overcome via treatment with protein synthesis inhibitors, which down-regulate expression of MCL-1. Thus, our findings may be similar to those found by Klanova et al. in diffuse large B- cell lymphoma, where the combination of homoharringtonine (parent compound of omacetaxine) and ABT-199 was active^52^. This group hypothesized that the efficacy of combination treatment is due to targeting both BCL-2 and MCL-1^53,54^. We confirmed decreased MCL-1 levels upon treatment with omacetaxine in the MDS-L cell line (Supplementary fig. 8E).

Importantly, neither the combination of omacetaxine and ABT-199 or omacetaxine and azacitidine had a significant effect on normal primitive cells in vitro. There are no significant changes in CD34+ viability of mobilized normal peripheral blood (Fig. 5c) and little toxicity in CD34+/CD38− cells (Supplementary fig. 8D).

Taken together, the data from in vitro culture of primary MDS specimens with varying drug combinations indicates that inhibition of both protein translation and oxidative phosphorylation mediates effective targeting of MDS stem cells.

### Xenograft modeling to target MDS stem cell metabolism

**Fig. 6:**
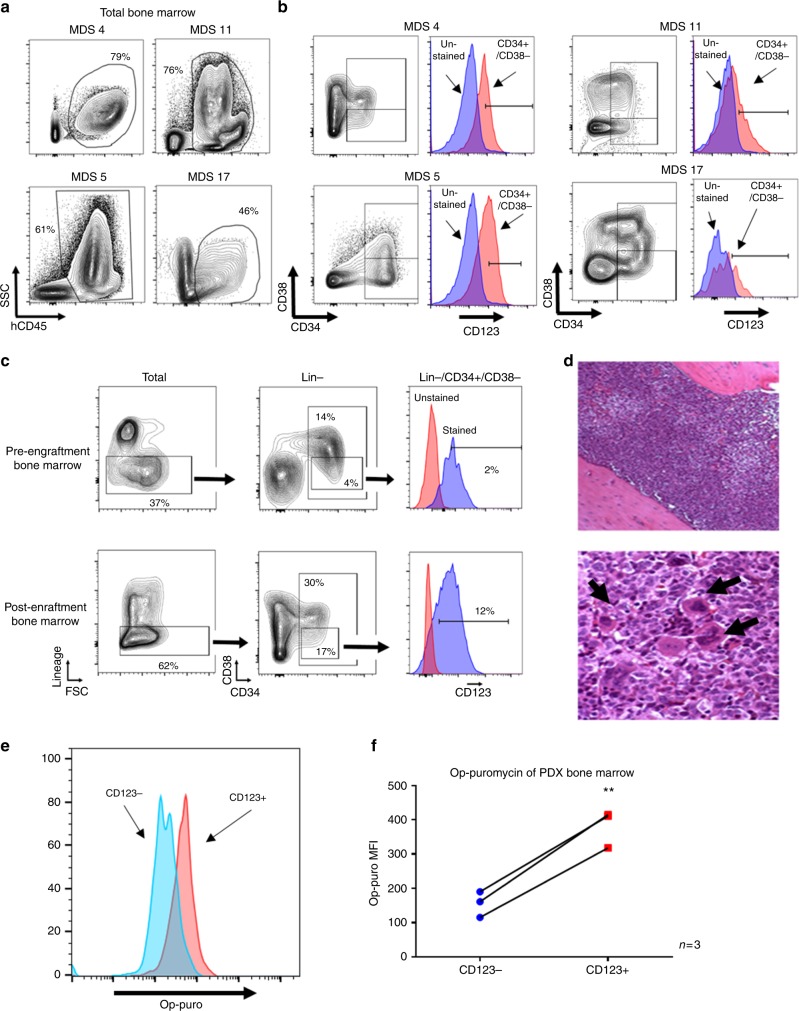
In vivo engraftment of high-risk primary MDS bone marrow specimens. Representative plots from 4 independent high risk MDS patient specimens engrafted in mice showing human CD45 staining (**a**), CD34/CD38 staining and CD123 staining (**b**). **c** Flow cytometry to analyze the immunophenoype of bone marrow specimens pre- and post-engraftment in NSG-S mice. **d** H&E staining of mouse femur post engraftment shows an abundance of blast-like cells as well as hypolobated megakaryocytes (arrows), indicative of acute disease evolving from antecedent MDS. **e** Representative plot of OP-puromycin labeling on cells derived from the marrow of xenograft mice. Labeling shown for CD123+ vs. CD123- cells, gated on CD45+/Lin−/CD34+/CD38−. **f** Aggregate data showing three independent patient xenografts stained for OP-puromycin as in panel **e**. ** indicates *p* < 0.01 (two-tail *t*-test) error bars are S.D

To further evaluate the clinical potential of various agents for targeting of MDS stem cells we developed an in vivo patient bone marrow derived xenograft model of MDS. The system employs the use of primary MDS specimens transplanted into immune deficient NSG-S mice. Engraftment of MDS specimens is generally challenging. In most cases, only very low levels of engraftment are possible. However, in the context of high-risk disease we have been able to achieve highly significant engraftment (~50–80% of bone marrow cells). Figure 6a shows representative plots of the human CD45 staining of mouse marrow post-engraftment from four separate primary human MDS specimens. The immunophenotype of these four specimens is further analyzed in Fig. 6b with respect to CD34/CD38 and CD123 expression. Shown in Fig. 6c is an analysis pre- and post-transplant for a primary human MDS specimen. Genotyping of marrow cells isolated post-transplant (analyzed in total human cells as well as lineage- and CD34+/CD123+ populations) show an allelic frequency for a diagnostic IDH2 mutation at 39.8–43.0%, virtually identical to the allele frequency in the pre-transplant specimen (43.06%)(Supplementary fig. 9A). These data indicate that the malignant cells are effectively maintained in the xenograft environment. Subsequent phenotypic analysis shows that the CD123+ subpopulation of MDS/AML stem cells demonstrates more efficient and robust engraftment of NSG-S mice, indicating that CD123+ cells represent a more aggressive or AML-like stage of disease. Specifically, the Lin- compartment increases about 2-fold, the more primitive compartment (Lin−/CD34+/CD38−) increases approximately 4-fold (4.4–16.6%) and the CD123+ subpopulation of the stem cell population increases approximately 7-fold (1.6–12.3%) in comparing pre to post engraftment cell populations (Fig. 6c). Histological analysis of the marrow of engrafted mice shows residual evidence of MDS-like cells, but predominantly a blast-like phenotype, consistent with transition to more malignant disease (Fig. 6d)^55^. Lastly, examination of protein translation in human CD45+/Lin−/CD34+/CD38−/CD123+ MDS stem cells (isolated post-engraftment) shows a significant increase over the human CD45+/Lin−/CD34+/CD38−/CD123− cells (Fig. 6e, f). We also find similar differential translation rates of mature cells in the patient xenografts to lin+ cells in patient specimens (supplementary fig. 9C). The robust engraftment, immunophenotype, mutational background, and blast-like phenotype are very similar to previously described AML xenograft studies, and we propose that our findings represent a model of a transitional stage of pathogenesis that spans advanced MDS and possibly extends to early AML-like disease.

**Fig. 7:**
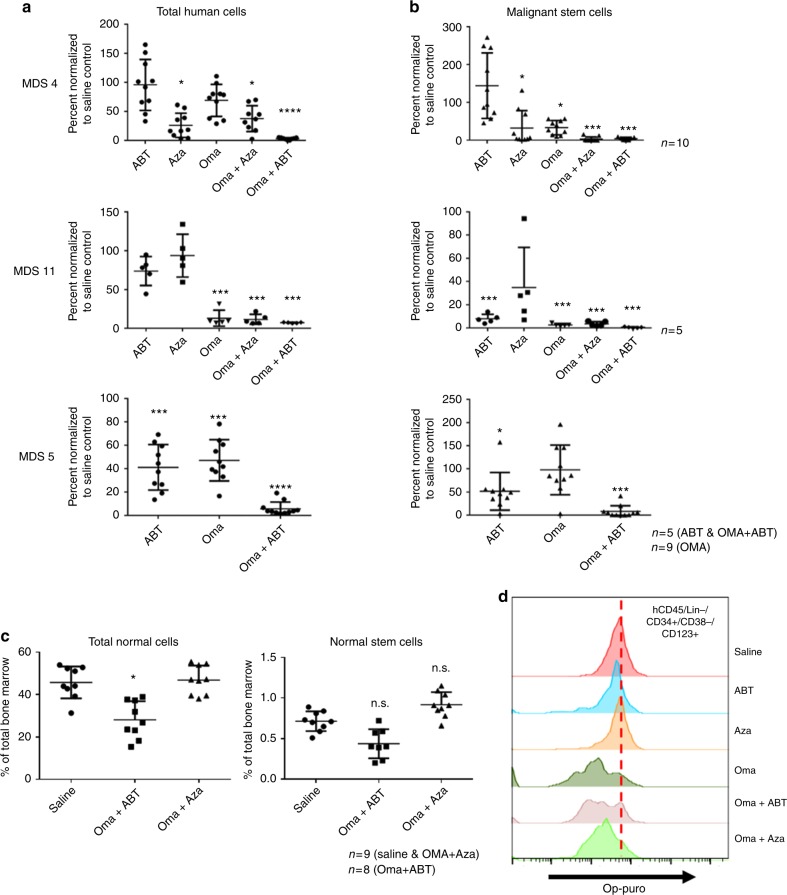
In vivo treatment of primary human MDS xenografts with omacetaxine mepesuccinate, ABT-199, and azacitidine. High-risk MDS patient bone marrow samples from three independent patients (**a**, **b**) or normal human CD34+ cells (**c**, **d**) were used to establish xenografts in NSG-S mice. After engraftment (~8 weeks post-transplant), animals were dosed daily with omacetaxine (Oma) alone (0.75–1.0 mg/kg) or in combination with ABT-199 (ABT) (50–100 mg/kg) or azacitidine (Aza) (1.5–3 mg/kg) for two weeks. Following treatment, human cell burden was measured by flow cytometry using antibodies specific to human CD45, lineage cocktail, CD34, CD38, and CD123. Graphs reflect total numbers of positive cells normalized to vehicle control animals. **a** Bulk human MDS cells (hCD45+). **b** MDS stem cells (hCD45+/Lin−/CD34+/CD38-/CD123+). **c** Total normal human cells (hCD45+) and normal stem cells (hCD45+/Lin−/CD34+/CD38−). Graphs reflect numbers of positive cells normalized to vehicle control animals or percentage of total bone marrow. * indicates *p* < 0.05; *** indicates *p* < 0.001 (*t*-test) error bars are S.D. **d** Representative OP-puromycin labeling of xenograft bone marrow cells following treatment with single and combination agents (gated on the hCD45+/Lin−/CD34+/CD38−/CD123+ population)

The establishment of a robust patient tissue derived xenograft allowed us to pre-clinically test the agents found to selectively target CD123+ cells in vitro. To this end, we employed xenografts established using specimens from three independent high-risk MDS patients (Fig. 7a, b). Drug treatments included single agent therapy with omacetaxine, ABT-199, and azacitidine and combination of omacetaxine with either ABT-199 or azacitidine. To evaluate overall drug activity, we first examined reduction of total human hematopoietic cells using the CD45 (pan-hematopoietic) antigen as a marker (Fig. 7a). We observed single agent toxicity of omacetaxine for human CD45+ cells in 2 of 3 patients. Single agent toxicity of ABT-199 and azacitidine for human CD45+ cells was also observed in 1 of 3 patients. Next, we examined drug activity in the stem cell compartment by gating on the CD34+/CD38−/CD123+ subpopulation (Fig. 7b). Notably, single agent toxicity of omacetaxine was observed for 2 of 3 patients in the more primitive cells. Each xenograft had a greater than or equal to 50% reduction in CD34+/CD38−/CD123+ cells with one patient having more than an 80% reduction in these cells. Thus, omacetaxine shows considerable single agent activity, when analysis is performed specifically within the primitive population. Interestingly, an examination of translation rates shows the xenograft with the least single agent efficacy correlates to the lowest translation rate in the MDS stem cells upon engraftment. The protein translation rate as measured by op-puro from CD123+ isolated from xenografted mice was ~25% less in the single agent insensitive patient xenograft (Fig. 6f). Significantly improved targeting was achieved using a combination of omacetaxine and ABT-199 or azacitidine, where effective elimination of both hCD45+ and CD34+/CD38−/CD123+ cells was observed (3 of 3 patients responded to the omacetaxine + ABT-199 combination, and 2 of 2 patients responded to the omacetaxine + azacitidine combination). Notably, in patient xenograft MDS4 the combination overcame the low single agent toxicity to eliminate the CD34+/CD38−/CD123+ cells almost completely. The potent eradication of MDS stem cells is in stark contrast to a lack of significant toxicity in parallel xenograft studies performed using normal human CD34+ cells. As shown in Fig. 7c, animals treated with either a combination of omacetaxine and azacitidine or omacetaxine and ABT-199 show very little effect in bulk cells and no significant effect in the CD34+/CD38− cells. To further explore the slight reduction in total CD45+ cells, we examined total CD34+ cells as well as CD19+ cells (B cell lineage) and CD33+ cells (myeloid lineage) (supplementary fig. 10A). For the omacetaxine and ABT-199 regimen, we detect approximately two-fold reductions in all compartments tested. For omacetaxine and azacytidine, there is no significant loss of CD34+ cells, and only a slight loss of CD19+ cells. A small enrichment in CD33+ cells suggests a selection for myeloid cells over the 2 week treatment (supplementary fig. 10A). In support of protein synthesis as the central mechanism of cell death, we observe that residual MDS cells recovered from treated MDS xenograft mice demonstrate reduced labeling with OP-puro (Fig. 7d). The toxicities found in normal xenografts correlate with translation rates of normal BM (supplementary fig. 10B). We find decreased translation rates in CD34+/CD38− cells and see no significant toxicity to either drug regimen in vivo. We see increased translation in the lin+ and lin−/CD34+ in normal BM and a corresponding small increase in toxicity for these populations in the normal xenografts (supplementary fig. 10).

## Discussion

Approximately 30% of high-risk MDS patients will progress to AML, making therapies that can suppress malignant populations at relatively early stages of disease evolution an appealing strategy. While the pathogenic progression from low-risk to high-risk MDS and ultimately to AML is well described, relatively little is known about evolution of the malignant stem cell population. Moreover, no clinical strategies for targeting the MDS stem cell population have been described, despite the clear potential of eradicating malignant stem cells prior to the onset of frank AML. Central to developing improved therapies is a closer examination of the primitive cells involved in the initiation/pathogenesis of MDS and understanding the key molecular events that drive disease progression.

In the present study, we investigated the phenotype and molecular properties of MDS stem cells of high-risk MDS patients. We examined patient bone marrow specimens with respect to immunophenotype, gene expression, cell biology, metabolic properties, and drug sensitivity. Our findings indicate that expression of CD123 within the stem cell population (CD34+/CD38−/Lin−) provides a powerful tool to identify and separate more advanced stages of malignancy from either normal stem cells or malignant stem cells at earlier stages of pathogenesis. The expression level of CD123 in the stem cell population was variable, but readily detected in all high risk MDS patients tested and represents a unique identifier of late pathogenic events as MDS transitions to AML. The expression of CD123 has previously been shown to be a highly consistent property of AML LSCs and has been studied as a phenotypic marker and therapeutic target^16,47,56^.

Our initial molecular analyses investigated transcriptomic properties of CD123+ stem cells. The comparison of Lin−/CD34+/CD38−/CD123+ to the bulk specimen and Lin−/CD34+/CD38−/CD123− showed several distinguishing characteristics; however, by far the most prevalent was activation of pathways associated with protein synthesis. This is an intriguing finding, particularly given recent studies demonstrating a central role for protein synthesis levels in the control of self-renewal and differentiation in normal HSCs^19^. While upregulation of protein synthesis has previously been described for both solid tumors and leukemia^57–60^, the role of protein synthesis in malignant stem cells is not well described. One study by Majeti et al. reported increased expression of ribosome pathway genes in AML stem cells, consistent with our findings in MDS^61^. Thus, the hypothesis that protein synthesis increases upon transition to AML is in good agreement with previous AML studies. Further, a previous study by McGowan et al. has shown that in comparison to normal HSCs, the MDS stem cell population displays reduced expression of ribosomal subunits^62^. This finding suggests the intriguing hypothesis that myeloid disease progression is characterized by an initial reduction in protein synthesis (during early to mid stages of MDS), manifesting dysfunction in marrow cell growth, subsequently followed by a re-activation of protein synthesis as a requisite step in progression to AML. Taken together, these findings indicate that a better understanding of protein synthesis as a regulator of overall stem cell physiology and metabolism in MDS/AML may provide new opportunities for therapeutic intervention.

Despite the clear increase in protein synthesis observed for CD123+ MDS stem cells, we did not detect increased cell proliferation. This somewhat counterintuitive finding is consistent with previous studies in AML, where the LSC population has been reported to be mostly quiescent^12,63^. The quiescent nature of CD123+ MDS stem cells suggests that elevated protein synthesis must be playing a role in physiological processes other than cell division. Indeed, our metabolomic analysis indicates profound changes in several intracellular processes, including energy metabolism and oxidative balance. Specifically, we see increased utilization of oxidative phosphorylation. Our previous work in AML demonstrated that LSCs are uniquely reliant on oxidative phosphorylation for survival. Hence increased oxidative phosphorylation activity as cells transition from MDS to AML is consistent with our AML studies. Furthermore, reports in both melanoma and pancreatic cancer have shown a preferential reliance on oxidative phosphorylation for survival of malignant stem cells, suggesting that this fundamental aspect of energy metabolism may be a broadly shared property of other neoplasms^43,44^.

Our work has also shown that Bcl-2 inhibitor drugs function to rapidly inhibit oxidative phosphorylation, prompting us to further evaluate toxicities previously found with such agents in MDS^64^. The data indicate that CD123+ MDS stem cells are impaired by Bcl-2 inhibition. Bringing the protein synthesis and metabolism findings together, we demonstrate selective and potent eradication of MDS stem cells using both cultured primary cells and xenograft models. Importantly, combination of drugs that inhibit protein synthesis (omacetaxine) and oxidative phosphorylation (omacetaxine & ABT-199) appears to be additive to synergistic, without increased toxicity to normal HSCs, including in vivo. The agents employed for our preclinical studies are currently approved for human use, thereby providing immediate opportunities for clinical translation and the design of trials aimed at directly targeting the most malignant elements of the MDS stem cell population. Using either ABT-199 or omacetaxine in combination with azacitidine is a particularly appealing strategy, since 5-azacitidine already has proven utility for at least some MDS patients^65^. Thus, it may be possible to design strategies that combine both direct targeting of MDS stem cells and the suppression of bulk disease while sparing normal HSCs.

In conclusion, our studies describe key molecular events that occur during the progression of late stage MDS to AML. The findings provide the ability to physically isolate and study a key step in pathogenesis and to develop new therapeutic strategies that may ultimately yield improved clinical outcomes.

## Methods

### Human specimens

MDS specimens were obtained from bone marrow of patients who gave informed consent for sample banking at the University of Colorado (COMIRB #12-0173 and 06-0720). Normal bone marrow and normal cord blood specimens were obtained from volunteer donors who gave informed consent on a research review board-approved protocol at the University of Colorado. Total MNCs were isolated from normal bone marrow or normal cord blood donor specimens by standard Ficoll procedures as previously done^56^ (GE Healthcare). See Supplementary Table 3 for additional details on human specimens.

### Cell culture and media

Primary cells were cultured as previously done in Pei et al.^66^. Primary human AML cells, normal cord/peripheral blood mononuclear cells (CBMCs/PBMCs) were resuspended at about 100–200 e^6^ cells per ml in freezing media composed of 50% FBS (GE Healthcare), 10% DMSO (Sigma) and 40% IMDM media (Gibco) and then cryo-preserved in liquid nitrogen. Cells were thawed in 37 °C water bath, washed twice in thawing media composed of IMDM (Gibco), 2.5% FBS (GE Healthcare) and 10 ug/ml DNAse (Sigma). Normal CD34+ CBMCs/PBMCs were enriched from CBMCs/PBMCs using the CD34 MicroBead kit (Miltenyi Biotec). Cells were cultured in normal or complete serum-free media (SFM) in 37 °C, 5% CO_2_ incubator. Normal SFM is composed of IMDM (Gibco), 20% BIT 9500 (STEMCELL Technologies), 10ug/ml LDL (Low Density Lipoprotein, Millipore), 55uM 2-Mercaptoethanol (Gibco) and 1% Pen/Strep (Gibco). Complete SFM were made by supplementing the normal SFM with FLT-3, IL-3 and SCF cytokines (PeproTech), each at 10 ng/ml. MDS-L cells were a generous gift from Daniel Starczynowski and grown in RPMI-1640 medium with 10% FBS, 1% penicillin-streptomycin and supplemented with 10 ng/ml IL-3^67^.

### Flow cytometry

After each treatment, cells were washed with ice-cold FACS buffer (PBS with 0.5% FBS) and then stained for 15 min at 4 °C in FACS buffer containing antibodies against human CD34 (BD Biosciences), CD38(BD Biosciences), Lineage cocktail (BD Biosciences), CD123(BD Biosciences), CD90, (BD Biosciences), CD45(BD Biosciences), and Near IR live dead (Invitrogen). The measurements of intracellular proteins were assayed per the Fix Perm kit (BD Biosciences) and stained for RPL5 (ImageBio). For measurement of ROS, CellRox (Invitrogen) was used to measure per manufacturer's protocol. Stained cells were analyzed immediately on a FACS Celesta cytometer (BD Biosciences). In order to isolate specific subpopulations, the cells were stained as above and a FACS Aria II (BD Biosciences) was used to viably sort cells for further assays. Data were analyzed and prepared via FlowJo^TM^ (Treestar).

### Droplet digital PCR

Bone marrow or peripheral blood samples were either lysed whole or Ficoll-separated and the mononuclear cell population lysed for DNA. Genomic DNA extraction was performed using the QIAamp DNA Mini kit (Qiagen), and quantity and purity were measured via Nanodrop.

Droplet digital PCR assays for common AML-associated mutations were designed either by our lab or obtained pre-designed from RainDance Technologies. These assays utilize common forward and reverse primers which span a single nucleotide polymorphism (SNP) of interest, as well as competitive TaqMan probes for wild-type and mutant alleles (labeled with VIC or FAM fluorophores, respectively). All assays were validated via quantitative PCR as well as droplet digital PCR to ensure optimal annealing temperatures and minimal false positive reads, using no template controls and known wild-type DNA samples for reference. Limit of detection in our hands is 0.01% using 100 ng of DNA template, and 0.005% using 1 ug of DNA template.

For patient sample series, 100 ng of DNA was added to a PCR master mix containing gene-specific primers and probes, droplet stabilizer, and ABI TaqMan Genotyping Master Mix (catalog number 4371355). PCR samples were loaded onto a RainDance Source chip, which was then inserted into the RainDance Source instrument to generate picoliter-size droplets containing individual molecules of DNA template. Dropletized samples were subjected to standard PCR using assay-optimized annealing temperatures. PCR-amplified samples were then placed into a droplet reader (RainDance Sense instrument) which counts individual droplets as “negative”, “FAM-positive,” or “VIC-positive.” Data was generated as.fcs files, which was then analyzed with RainDrop Analyst II software. Allele frequencies are expressed as the percentage of mutant (FAM)-positive droplets relative to the total number of positive droplets (FAM+ VIC).

### Mass cytometry

These samples were processed and stained as described in Amir et al.^68^. Briefly, frozen aliquots of cells were thawed in 98% IMDM + 2% FBS + DNase I. Thawed cell pellets were immediately re-suspended in PBS containing 5 µM Cell-ID Cisplatin (Fluidigm) and incubated for 5 min at room temperature. Cells were washed one time with cell staining buffer and fixed using 2% formaldehyde for 10 min at room temperature. Fixed cells were washed with cell staining media (low barium PBS + 0.5% bovine serum albumin + 0.02% sodium azide) and incubated for 30 min at room temperature in surface epitope metal conjugated antibodies (Supplementary Table 4). Surface stained cells were washed twice and re-suspended in cold methanol for 30 min at 4 °C. Methanol permeabilized cells were washed one time and re-suspended in intracellular epitope metal conjugated antibody mix for 1 h at room temperature. Stained cells were washed once before incubation in 0.5 µM Cell-ID Intercalator-Ir (Fluidigm). Fixed and stained cells were washed two times in cell staining media and re-suspended at (2.5–5) × 10^5^ /ml in water before being run on CyTOF instrument.

Data was analyzed utilizing the SPADE3 algorithm which was used to visualize and interpret data^69^. The analysis was carried out in MATLAB per the original references. The Auto Suggest Annotation of SPADE3 was utilized to decide clustering.

### Translation assay

In order to evaluate protein translation cells were stained as above and the OP-Puro Assay kit (Invitrogen) was used per the manufacturer instructions to evaluate translation. Briefly, cells were surface stained and incubated with OP-puromycin substrate, fixed, and Click-IT chemistry was used to evaluate levels of staining. Stained cells were analyzed immediately on a FACS Celesta cytometer (BD Biosciences). Data was analyzed and prepared via FlowJo.

### In vivo treatment of xenografts

Animal experiments were conducted in accordance with an approved protocol (IACUC #0308) at the University of Colorado. NSG-S mice were conditioned 24 h prior to transplant with 25 mg/kg busulfan via IP injection. MDS patient bone marrow mononuclear cells (0.8–1.0e^6^) were transplanted via tail vein in a final volume of 0.1 ml of PBS with 0.5% FBS. After 6–10 weeks based on engraftment levels, animals were separated to treatment and control groups (10 animals per group) and received either ABT-199 (50 mg/kg, oral gavage for 5 daily doses, 2 days off and 5 daily doses), omacetaxine (0.5 mg/kg or 0.75 mg/kg IP daily for 5 daily doses, 2 days off and 5 daily doses), azacitidine (1.5 mg/kg or 3.0 mg/kg IP every other day for 2 weeks) or vehicle. After treatment, mice were sacrificed and human engrafted cells were evaluated by flow cytometry, droplet digital PCR, and H&E staining.

### RNA-seq

The TruSeq RNA Sample Preparation Kit V2 (Illumina) was used for next generation sequencing library construction per the manufacturer’s protocols. Amplicons are ∼200–500 bp in size. The amplified libraries were hybridized to the Illumina single end flow cell and amplified using the cBot (Illumina). Single end reads of 100 nucleotides were generated for each sample and aligned to the organism specific reference genome. Raw reads generated from the Illumina HiSeq2500 sequencer were de-multiplexed using configurebcl2fastq.pl version 1.8.4. Quality filtering and adapter removal was performed using Trimmomatic version 0.32 with the following parameters: “SLIDINGWINDOW:4:20 TRAILING:13 LEADING:13 ILLUMINACLIP:adapters.fasta:2:30:10 MINLEN:15”. Processed/cleaned reads were then mapped to the UCSC hg19 genome build with SHRiMP version 2.2.3 with the following setting: “–qv-offset 33 -all-contigs.”

### Extraction of metabolites and metabolomic analysis

Sorted MDS patient bone marrow cells were pelleted and immediately extracted in ice-cold lysis/extraction buffer (methanol:acetonitrile:water 5:3:2) at 2 million cells per ml. Samples were then agitated at 4 °C for 30 min and centrifuged at 10,000 × *g* for 15 min at 4 °C. Protein and lipid pellets were discarded, whereas supernatants were stored at −80 °C before metabolomic analyses. Metabolomic analyses were performed as previously reported^66^. Analyses were performed using a Vanquish UHPLC system (Thermo Fisher Scientific, San Jose, CA, USA) coupled online to a Q Exactive mass spectrometer (Thermo Fisher Scientific, San Jose, CA, USA). Cell extracts (20 μl) we resolved using a 15 min method on an Acquity UPLC BEH Amide Column, 2.1 × 100 mm, 1.8 µm particle size (Waters, Milford, MA, USA) run at 350 µl/min and 35 °C (mobile phases—A: 5% acetonitrile, 10 mM ammonium acetate, pH 10.0; B: 95% acetonitrile, 10 mM ammonium acetate, pH 10.0 adjusted with MS grade ammonium hydroxide). The UHPLC system was coupled online with a Q Exactive system (Thermo Fisher Scientific) scanning in full MS mode (2 μscans) at 70,000 resolution in the 60–900 *m*/*z* range, 4-kV spray voltage, 15 sheath gas and 5 auxiliary gas operated either in negative and positive ion mode. Calibration was performed before each analysis against positive or negative ion mode calibration mixes (Thermo Fisher Scientific) to ensure sub-ppm error on the intact mass. Upon conversion of.raw files into.mzXML format using MassMatrix, metabolite, and isotopologue assignments were performed using the software Maven following the rationale described by Buescher et al.^70^. Assignments were further confirmed by chemical formula determination from isotopic patterns and accurate intact mass and retention times against over 650 standards, including commercially available glycolytic and Krebs cycle intermediates, amino acids, glutathione intermediates, and nucleoside phosphates (Sigma, IROATech).

### Statistical analysis

All statistical tests between two groups were done with GraphPad Prism software using unpaired two-tailed *t* test unless otherwise noted in legend.

## Electronic supplementary material

Supplementary Information

Peer Review File

## Data Availability

The data that support the findings of this study are available from the corresponding author upon reasonable request. RNA sequencing data that support the findings of this study have been deposited in GEO with the accession codes GSE116100.
