# Supplementary material for: Characterization and targeting of malignant stem cells in patients with advanced myelodysplastic syndromes
Source: Nat Commun. 2018 Sep 12;9:3694. doi: 10.1038/s41467-018-05984-x (PMC6135858; doi:10.1038/s41467-018-05984-x)
Supplement: Supplementary file 1 — Supplementary Information [file 41467_2018_5984_MOESM1_ESM.pdf]

# Characterization and Targeting of Malignant Stem Cells in Patients with Advanced Myelodysplastic Syndromes

Stevens et. al.

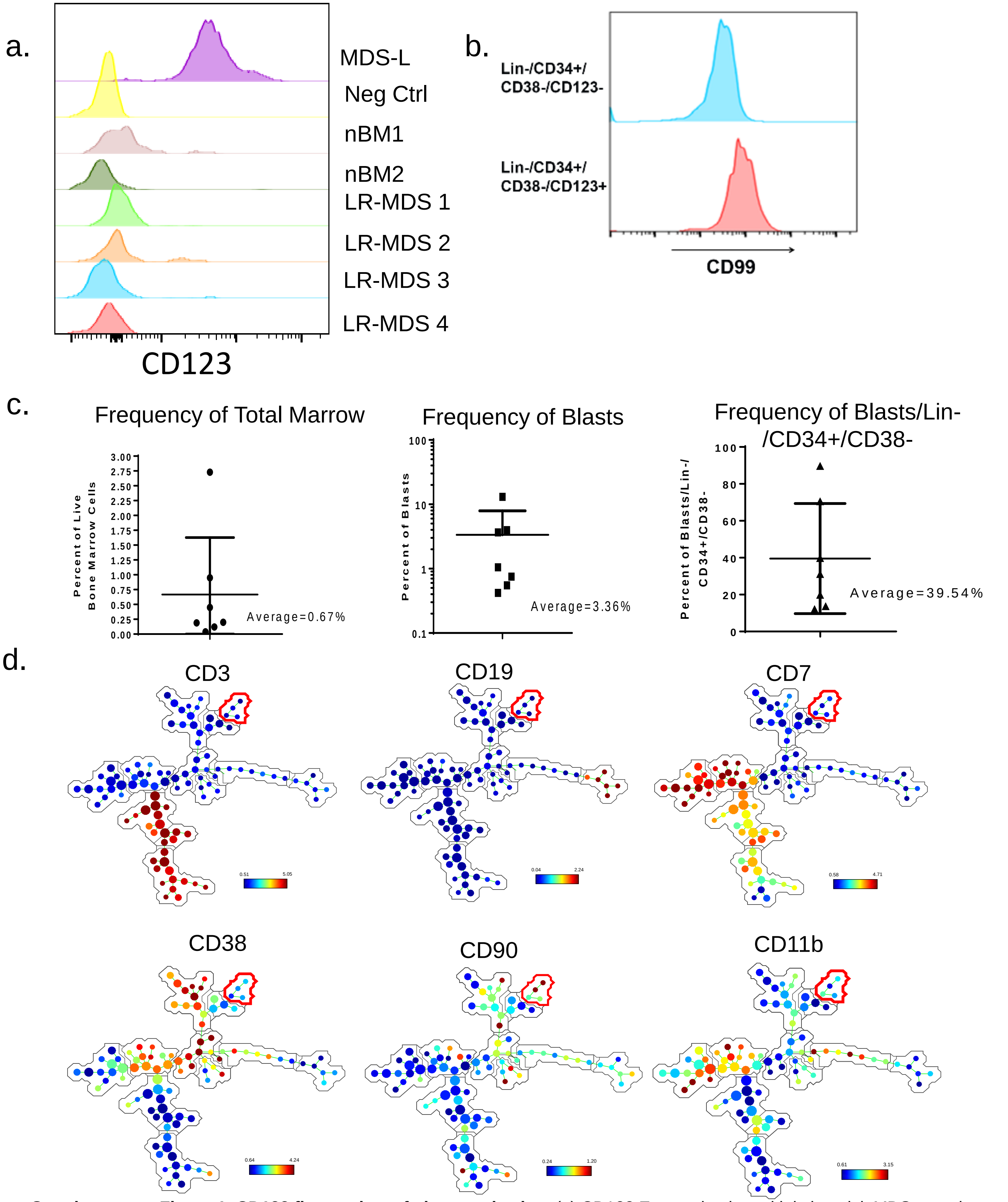


**Supplementary Figure 1. CD123 flow and cytof characterization.** (a) CD123 Expression in multiple low-risk MDS samples. Patient bone marrow specimens were stained for lineage markers, CD34, CD38, and CD123. Plots represent CD123 expression in the Lin-/CD34+/CD38- subpopulation. CD123 expression is negative in both normal BM and low risk MDS specimens. MDSL cell line shown as positive control. (b) Representative plot of CD99 Expression in high-risk MDS samples. Patient bone marrow specimens were stained for lineage markers, CD34, CD38, CD123, and CD99. Plots represent CD99 expression in the Lin-/CD34+/CD38-/ CD123+ vs CD123- subpopulation.(c) Frequency of Lin-/CD34+/CD38-/CD123+ cells in total bone marrow, blasts, and CD34+/CD38- subpopulations error bars are s.d. n=7(d) Heatmap of CyToF clustering surface phenotype of Spade clusters in Fig 1a including CD123+ cluster that expresses TIM3, HLA-DR, CD90 but not CD38.

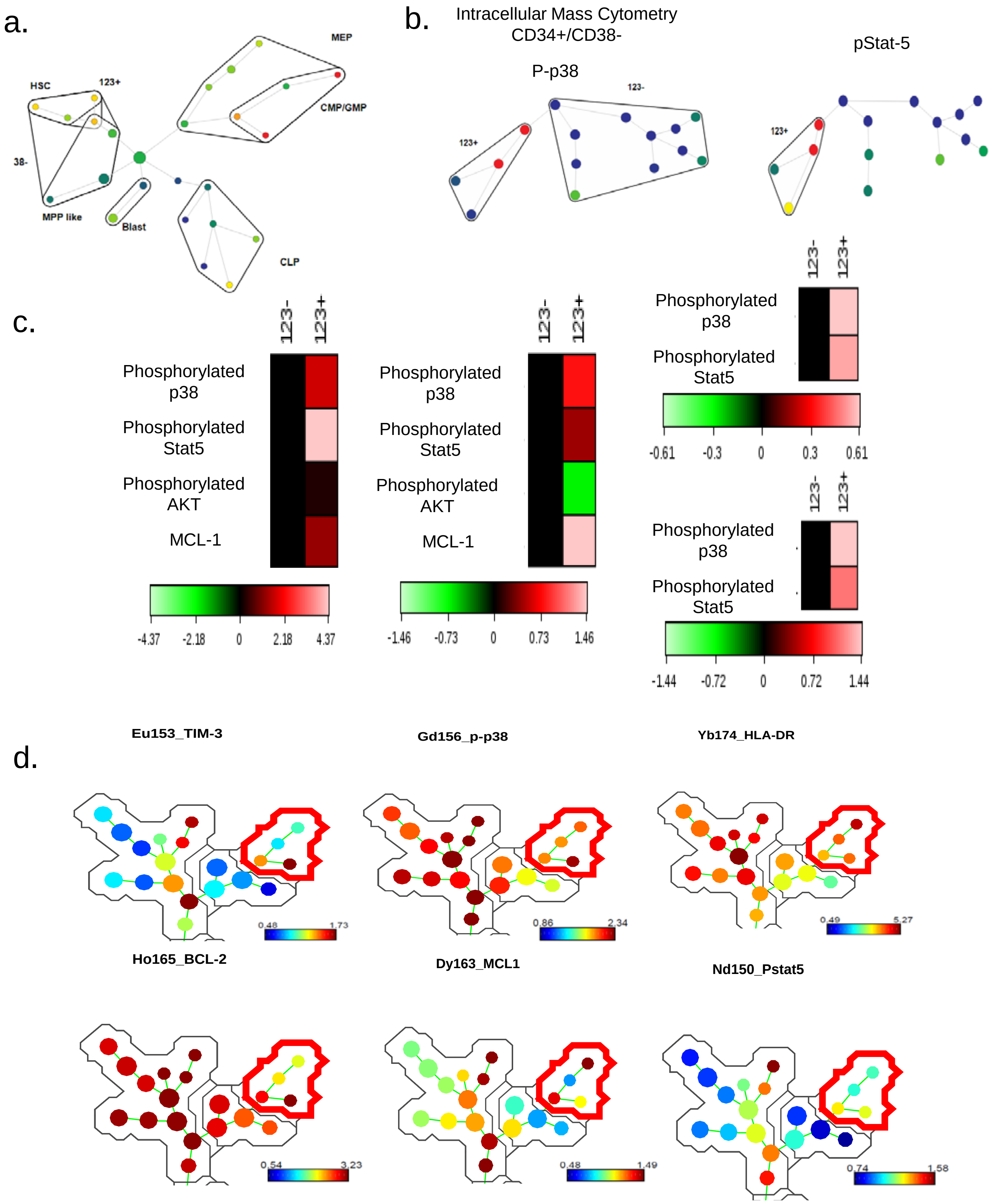


**Supplementary Figure 2. Intracellular signaling in high-risk MDS patient specimens.** Patient bone marrow specimens were labeled for mass cytometry and analyzed using the SPADE algorithm. (a) SPADE analysis of CD34+ population of MDS specimens grouping nodes by traditional immunophenotypic markers of stem and progenitor cells. Diagram is colored by CD123 expression.(b) Analysis of the CD34+/CD38- subpopulation for phosphorylated p38 (P-p38) and phosphorylated Stat5 (pStat5). Data is shown as the ratio of mean expression between Lin/CD34+/CD38- and CD123- vs. CD123+ subpopulations. (c) Relative levels of indicated signaling pathways in comparing the CD123- vs. CD123+ MDS stem cell populations. (d) Spade analysis of CD34+ subpopulations for intracellular and surface antigens from the same patient specimen shown in Figure 1.

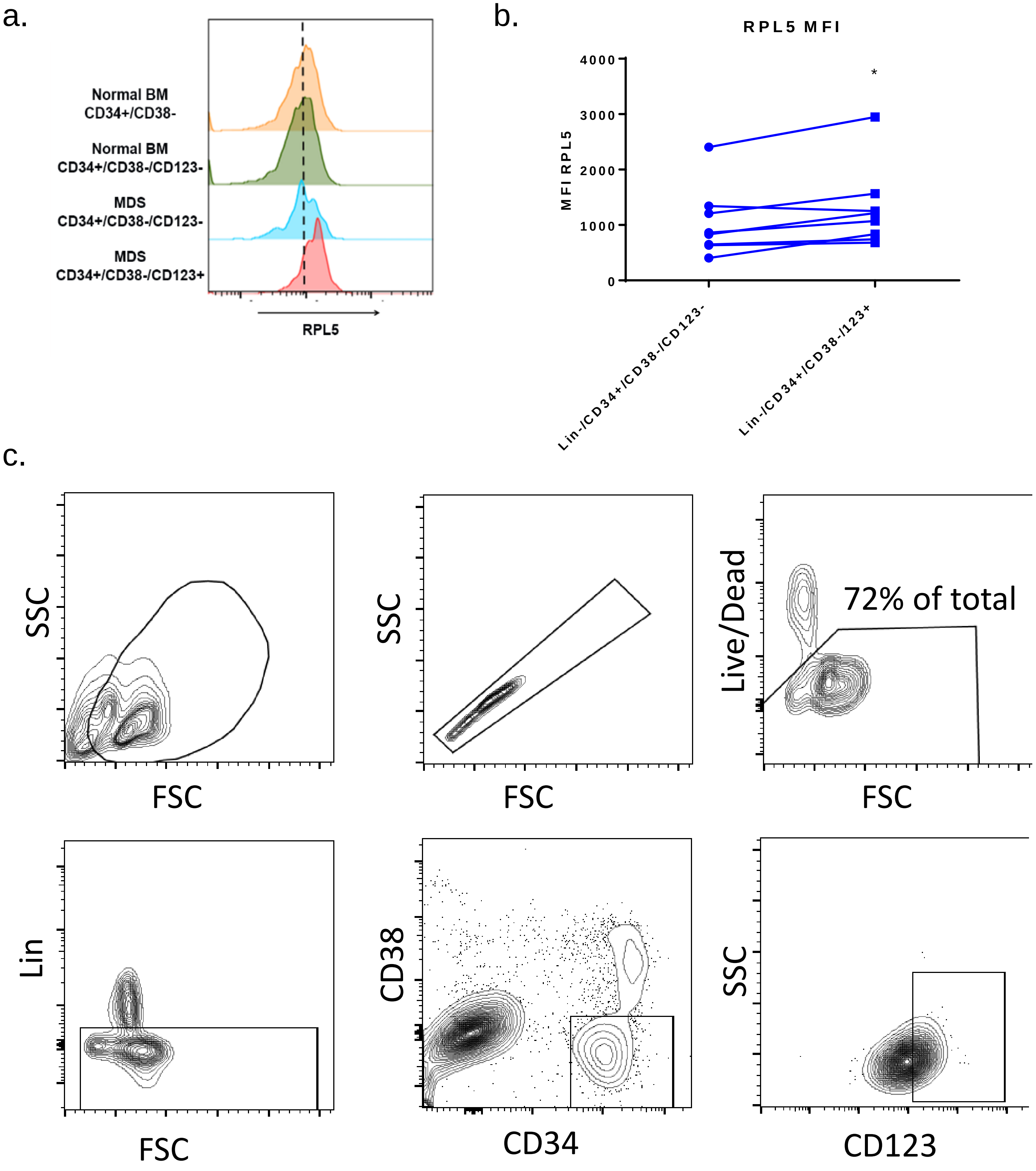


**Supplementary Figure 3. Representative flow staining of MDS patient specimens.**(a) RPL5 analysis by flow cytometry of a normal vs. a representative high-risk MDS specimen comparing Lin-/CD34+/CD38-/CD123+ vs CD123- subpopulations. The data shows a significant increase in CD123+ RPL5 mean fluorescent intensity. (b) Aggregate flow cytometry RPL5 expression data for 6 independent high-risk MDS specimens. *p<.05 (paired t-test) (c) Representative staining of cultured primary cells. Cell plots show analysis of stained control cells for in vitro drug dosing experiments.

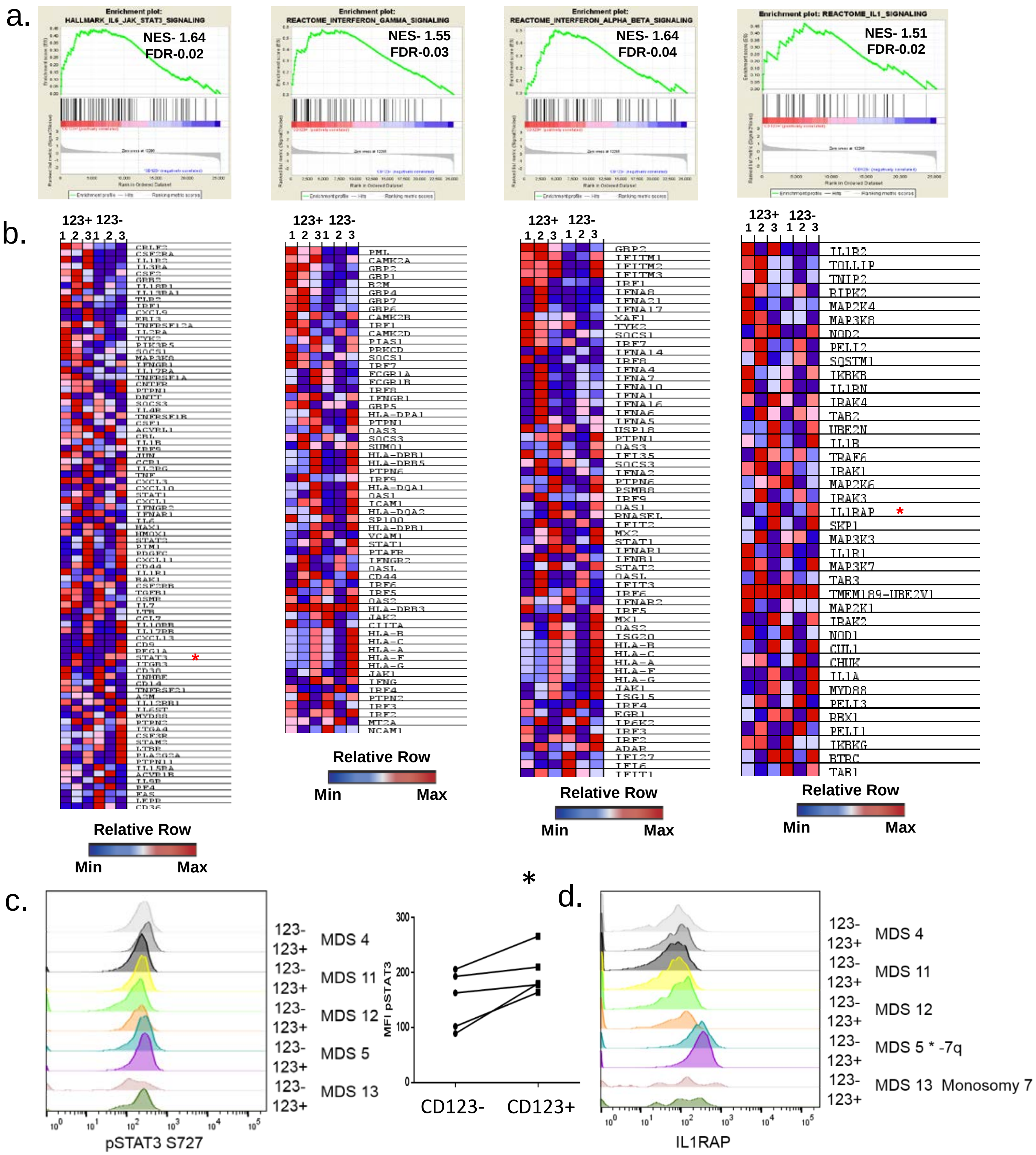


**Supplementary Figure 4. Whole transcriptome analysis shows an up-regulation of multiple pathways in CD123+ MDS cells.** RNA-seq was performed on Lin-/CD34+/CD38-/CD123 + or Lin-/CD34+/CD38-/CD123- primary MDS BM cells from three independent patients. (a) Using Enrichment map algorithm, top pathways and groups of pathways were delineated in CD123+ versus CD123- cells. Representative enrichment plots for gene sets in table 2 B and corresponding heat maps of gene expression from gene sets (b). (c) One of the most significantly enriched pathways shown in A and B was the STAT3 pathway. Analysis of 5 patient samples comparing Blasts/Lin-/CD34+/CD38/CD123+ versus Blasts/Lin-/CD34+/CD38-/CD123- for levels of phosphorylated STAT3 at Serine 727 shows a small but significant increase in CD123+ subpopulation phosphorylation. *<p.05 (t-test) n=5 (d) Increases in IL1 signaling found in gene set enrichment analysis are not due to IL1RAP expression. . Analysis of 5 patient samples comparing Blasts/Lin-/CD34+/CD38/CD123+ versus Blasts/Lin-/CD34+/CD38-/CD123- for levels of IL1RAP show elevation in chromosome 7 mutated specimens but no differential between CD123+ and CD123- n=5.

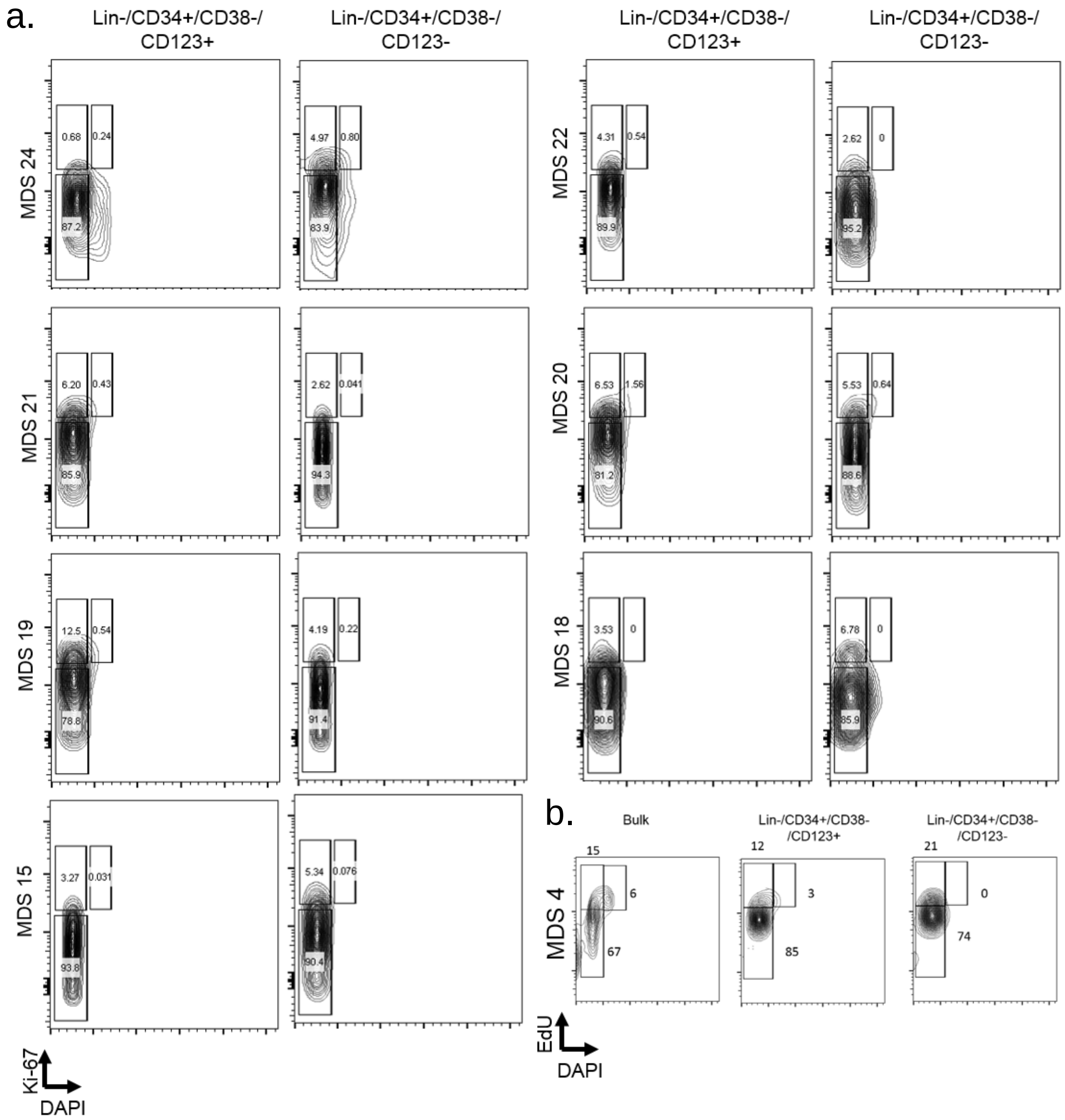


**Supplementary Figure 5. Cell cycle analysis of MDS patient specimens.** Cell cycle analysis of a representative MDS specimens from Figure 3D showing Blasts/Lin-/CD34+/CD38-/CD123+ vs Blasts/Lin-/CD34+/CD38-/CD123- stem cell populations stained for Ki67 and DAPI (a) or Ki67 and EdU (b).

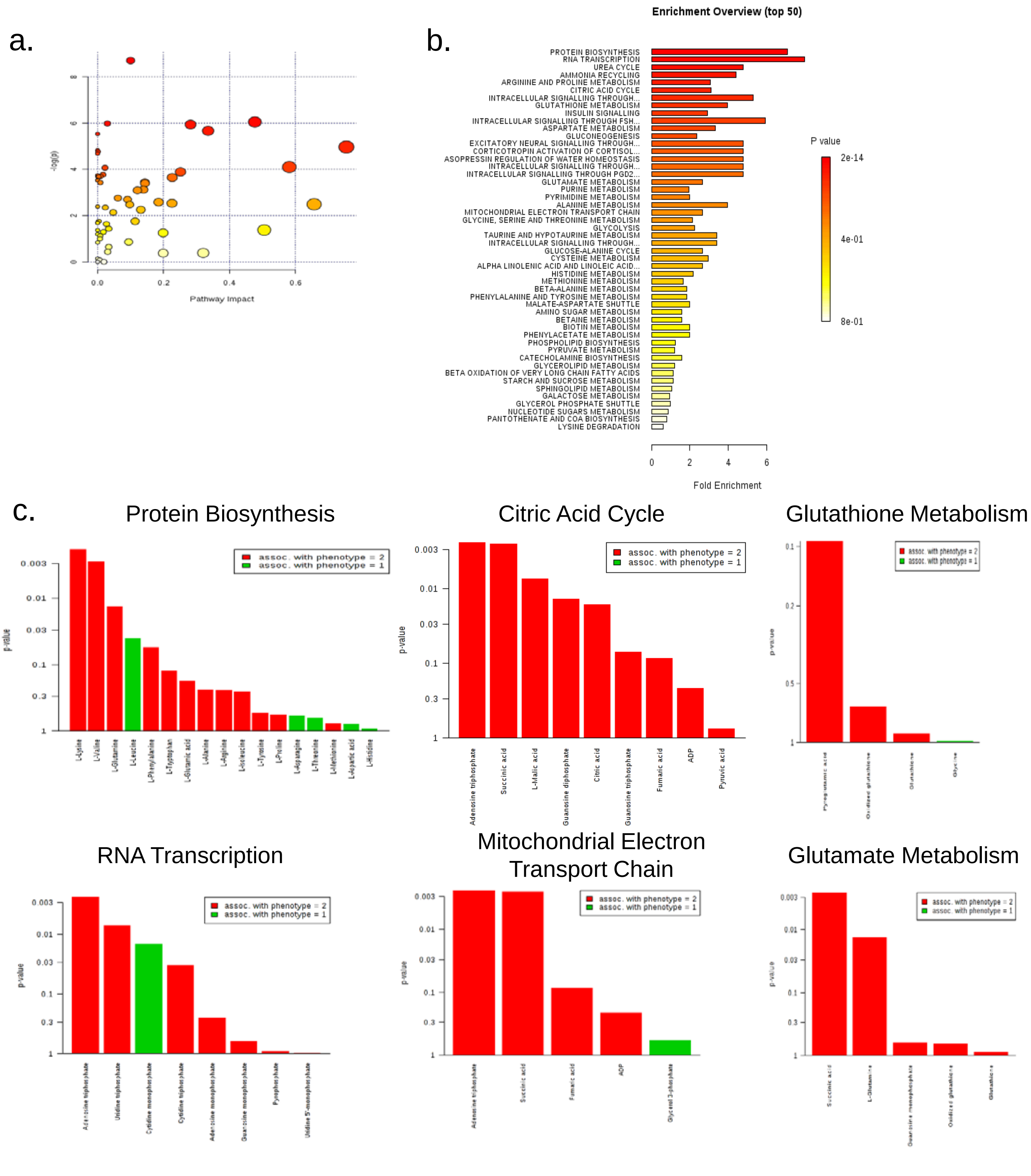


**Supplementary Figure 6. Lin-/CD34+/CD123+ cells exhibit a different metabolic phenotype than Lin-/CD34+/CD123- cells.**

MDS stem cells were isolated from patient marrow specimens and subjected to LC-MS metabolite analysis. (a-b) Comparisons of enriched pathways in Lin-/CD34+/CD123+ cells are shown. The fold enrichment by pathway impacted is plotted in panel a, where significance is shown on the vertical axis and metabolic impact (as assessed by Metaboanalyst software) is shown on the horizontal axis. Specific pathways enriched are shown in panel b. These data show increased translation and alterations in multiple metabolic pathways including energy metabolism (e.g. Citric Acid Cycle) and redox metabolism (e.g. Glutathione metabolism). (c) Individual metabolites contributing to pathway differences represented as p value of difference between CD123+(red) and CD123-(green).

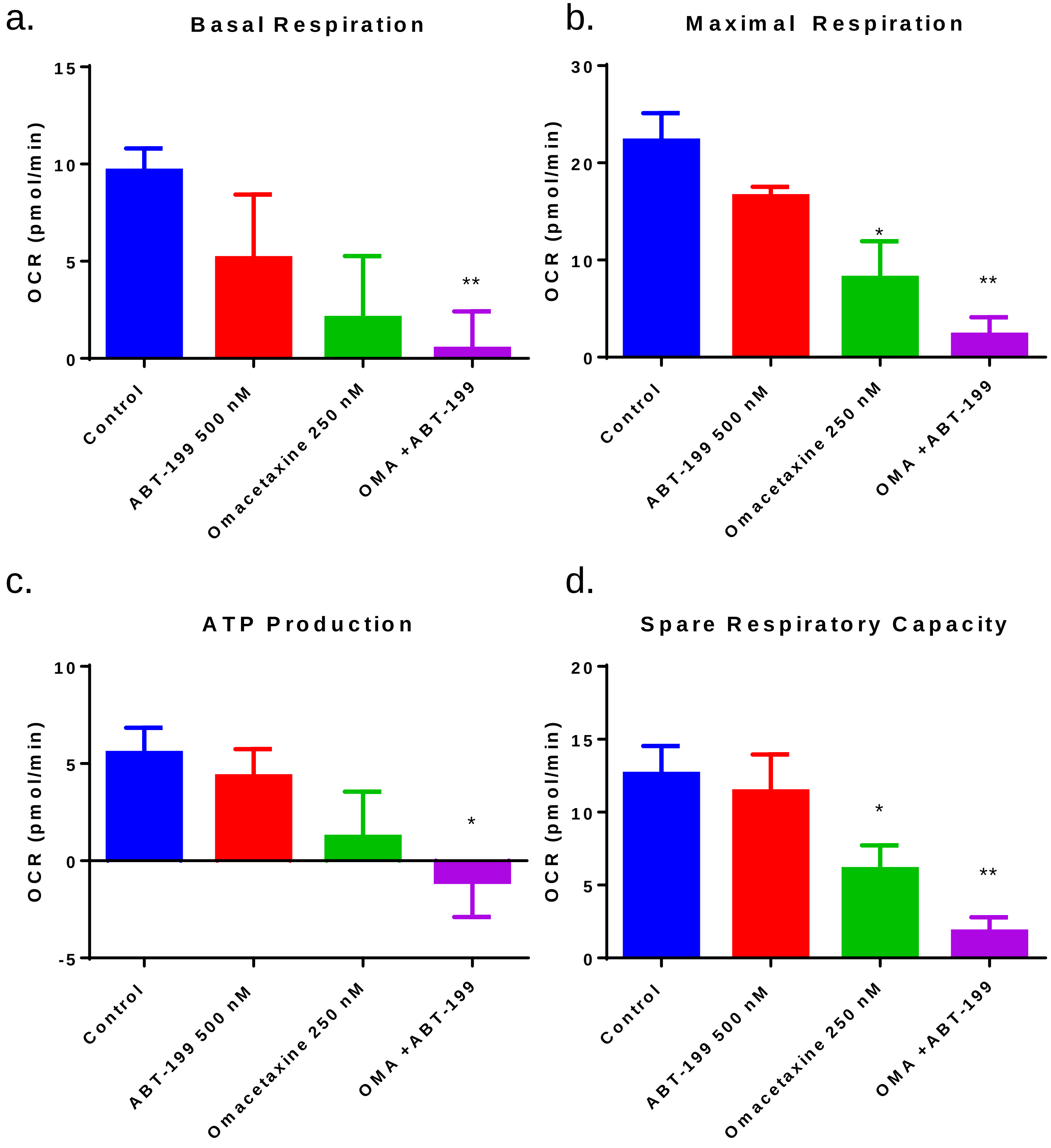


**Supplementary Figure 7. Effects of omacetaxine and ABT-199 on mitochondrial respiration in primary MDS blast cells.** MDS4 cells were sorted for blasts utilizing CD45+ and SSC and CD3-/CD19- and cultured in the presence of the following drugs for 4 hours and a mitochondrial flux assay was performed using the Agilent Seahorse XF96 instrument. The cells were dosed with ABT-199 500nM, omacetaxine (Oma) 250 nM, and omacetaxine 250 nM + ABT-199 500 nM. Omacetaxine in combination with ABT-199 causes significant decreases in OCR leading to a decrease in basal respiration (a), maximal respiration (b), ATP production (c), and spare respiratory capacity(d). * p<.05 and **<p.01 (t-test) error bars are s.d. n=5

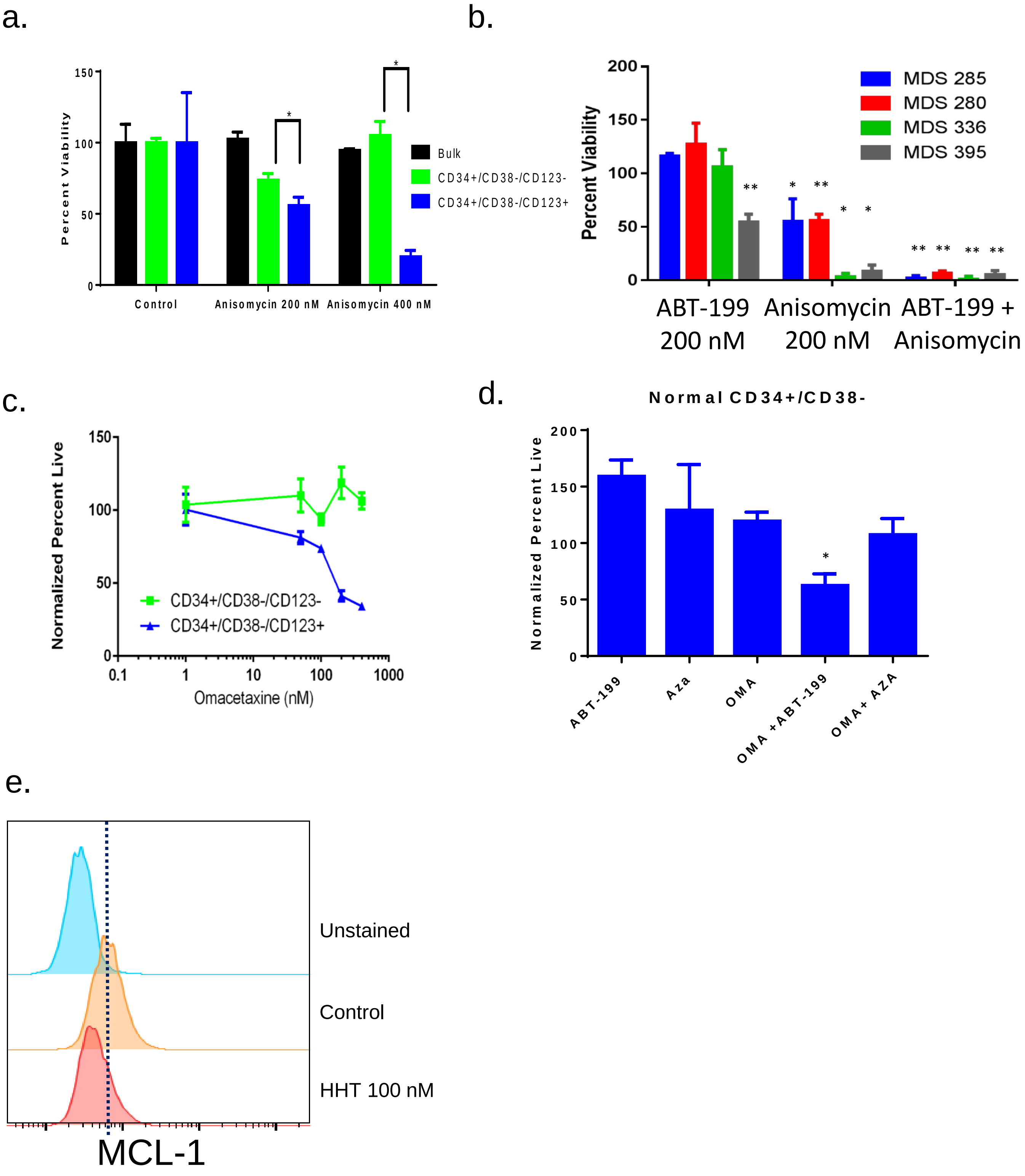


**Supplementary Figure 8. CD123+ cells are sensitive to protein translation inhibitors alone and in combination with ABT-199.** Patient bone marrow specimens were cultured in various concentrations of anisomycin and/or ABT-199 and stained for Lineage, CD34, CD38, and CD123. Graphs represent viability of each subpopulation (a) or CD123+ cells in the Lin-/CD34+/CD38- subpopulation of multiple patient samples (b). (c) CD123+ cells have increased sensitivity to Omacetaxine, a protein synthesis inhibitor, when compared with CD123- cells in the primitive compartment. (d) Normal primitive cells from mobilized peripheral blood (CD34+/CD38-) show little toxicity to single agent dosing and only moderate toxicity to the combination of Omacetaxine and ABT-199. (e) Homoharringtonine decreases MCL-1 expression at 100 nM in MDS-L cells after 4 hour incubation as measured by flow cytometry. .*<p.05, **<p.01(t-test) error bars are s.d. n=3

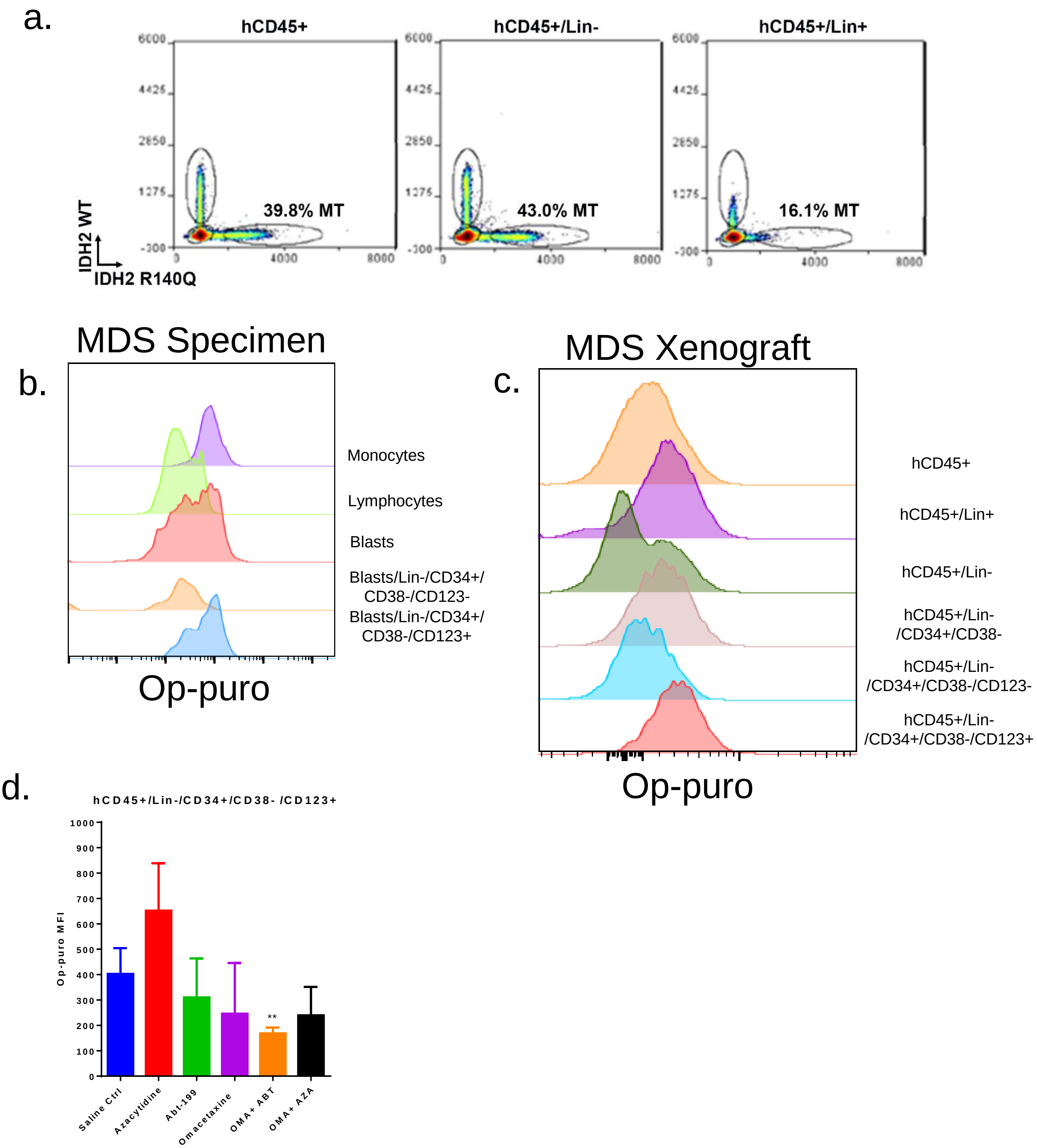


**Supplementary Figure 9. Characterization of Patient Xenografts** (a) Genetic phenotype of sorted human cells from NSG-S primary xenograft shows variant allelic frequencies are enriched in the lineage negative cells over the lineage positive cells.(b) Op-puro staining of subpopulation of primary xenograft subpopulations (c) OP-puro staining of bone marrow from MDS xenografts post in vivo treatment with indicated drugs. MFI shown for hCD45+/Lin-/CD34+/CD38-/CD123+ shows significant decreases in translation in the omacetaxine + ABT-199 group and trends of decreased translation in the omacetaxine and omacetaxine + azacitidine groups.(d) Normal cord blood xenografts treated with drug combinations of omacetaxine + azacitidine or omacetaxine + ABT-199 **<p.01 (t-test) error bars are s.d.

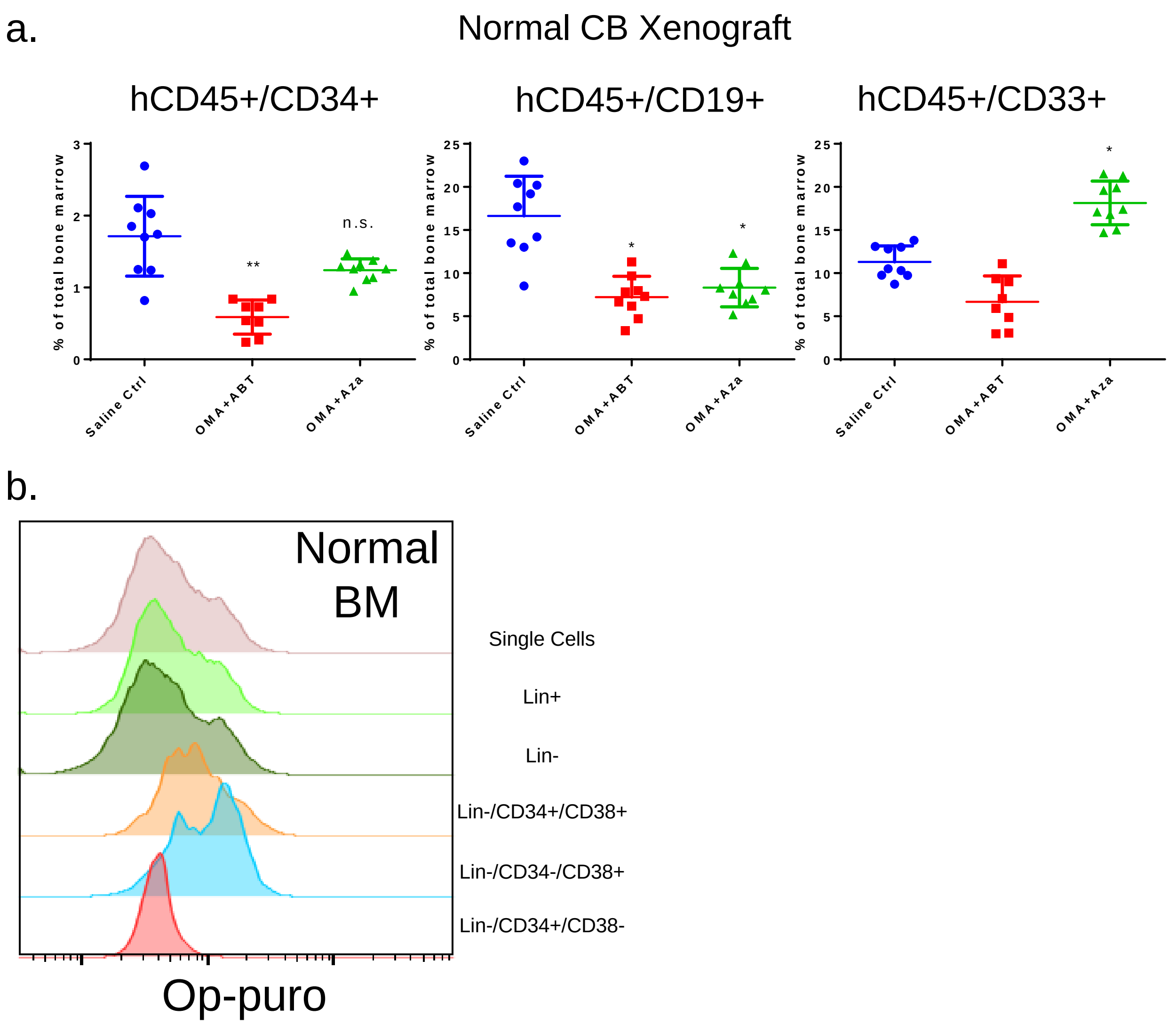


**Supplementary Figure 10. Characterization of normal cell xenografts and protein translation.** (a) Normal cord blood xenografts treated with drug combinations of omacetaxine + azacitidine or omacetaxine + ABT-199 and stained with markers of mature cells (CD19 & CD33) and stem and progenitors (CD34) (**<p.01 *<p.05 t-test) error bars are s.d. n=9 in saline and OMA+AZA and n=8 in OMA+ABT
(b) Op-puro staining of subpopulations of normal human bone marrow showing different translation rates in different subpopulations of cells.

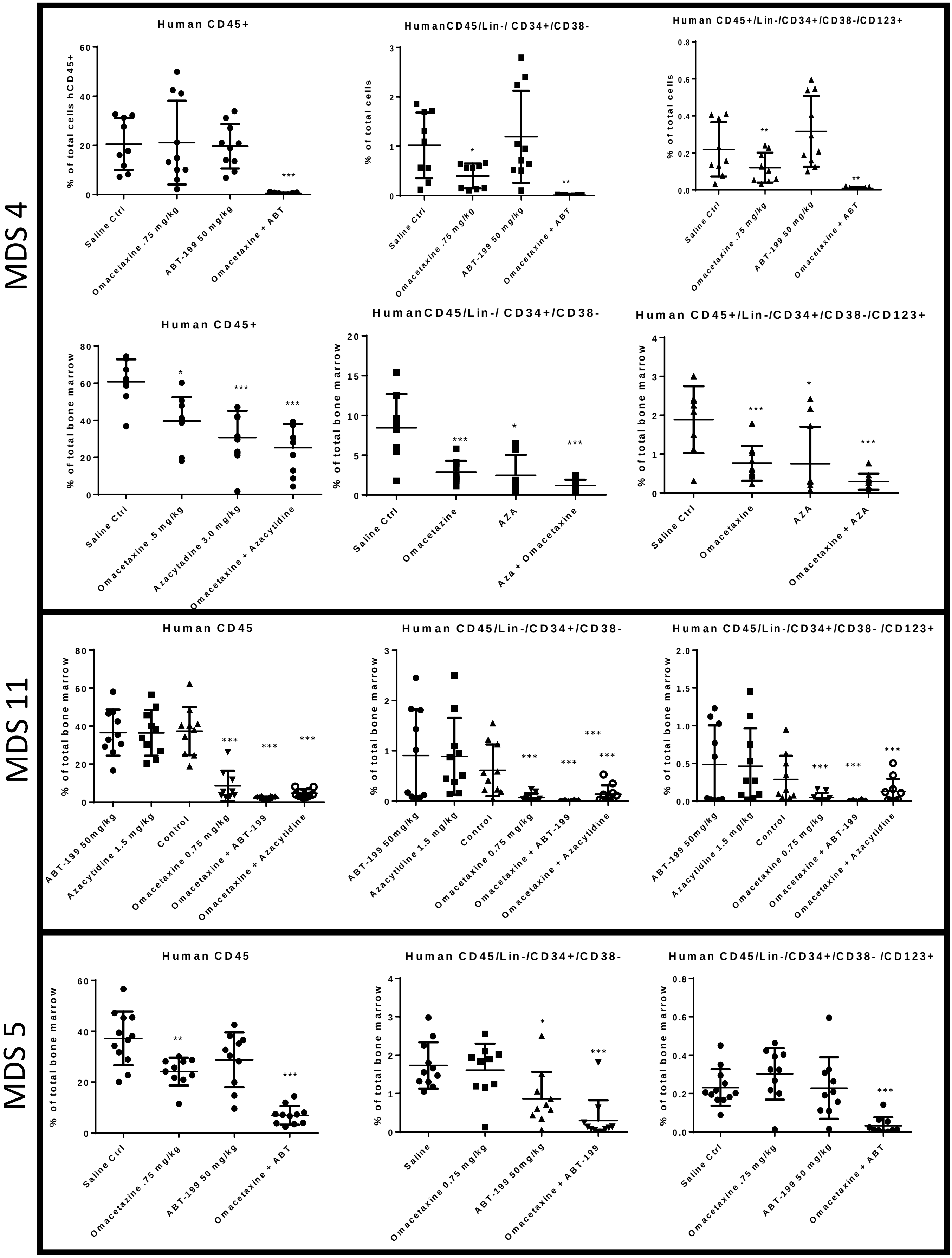


**Supplementary Figure 11. Raw percentages of total marrow for drug treated patient derived xenografts.** Values representing percentages of human CD45 of total marrow, human CD45/Lin-/CD34+/CD38- of total marrow, and hCD45+/Lin-/CD34+/CD38-/CD123+ of total marrow.*<p.05, **<p.01,***<p.001(t-test) error bars are s.d. n= 7-12 see main Figure

| Pathway | ES | NES | NOM p-val |
|---|---|---|---|
| KEGG_RIBOSOME | 0.73 | 2.97 | 0.000 |
| KEGG_RIG_I_LIKE_RECEPTOR_SIGNALING_PATHWAY | 0.45 | 1.77 | 0.000 |
| KEGG_REGULATION_OF_AUTOPHAGY | 0.52 | 1.77 | 0.002 |
| KEGG_SPLICEOSOME | 0.38 | 1.63 | 0.002 |
| KEGG_STEROID_BIOSYNTHESIS | 0.58 | 1.60 | 0.022 |
| KEGG_PRIMARY_BILE_ACID_BIOSYNTHESIS | 0.56 | 1.53 | 0.039 |
| KEGG_OLFACTORY_TRANSDUCTION | 0.30 | 1.49 | 0.000 |
| KEGG_CYTOSOLIC_DNA_SENSING_PATHWAY | 0.40 | 1.48 | 0.022 |
| KEGG_PROTEIN_EXPORT | 0.48 | 1.44 | 0.046 |
| KEGG_TASTE_TRANSDUCTION | 0.37 | 1.35 | 0.055 |
| KEGG_LONG_TERM_POTENTIATION | 0.34 | 1.31 | 0.081 |
| KEGG_ACUTE_MYELOID_LEUKEMIA | 0.33 | 1.23 | 0.138 |

**Supplementary Table 1. Top 10 KEGG Pathways enriched in Lin-/CD34+/CD38-/CD123+.** Gene set enrichment (GSEA) was performed using RNA-seq data from sorted Lin-/CD34+/CD38-/CD123+ vs. Lin-/CD34+/CD38-/CD123- cells. The top 10 hits from this analysis are shown in the table with KEGG_Ribosome being the most significant.

| | GS DETAILS | NES | NOM p-val |
|---|---|---|---|
| 1 | KEGG_SYSTEMIC_LUPUS_ERYTHEMATOSUS | -1.52 | 0.000 |
| 2 | KEGG_DRUG_METABOLISM_OTHER_ENZYMES | -1.52 | 0.005 |
| 3 | KEGG_PARKINSONS_DISEASE | -1.47 | 0.012 |
| 4 | KEGG_LYSOSOME | -1.44 | 0.029 |
| 5 | KEGG_SPLICEOSOME | -1.43 | 0.015 |
| 6 | KEGG_AUTOIMMUNE_THYROID_DISEASE | -1.40 | 0.056 |
| 7 | KEGG_PYRIMIDINE_METABOLISM | -1.38 | 0.039 |
| 8 | KEGG_GLUTATHIONE_METABOLISM | -1.35 | 0.063 |
| 9 | KEGG_ALLOGRAFT_REJECTION | -1.34 | 0.084 |
| 10 | KEGG_HUNTINGTONS_DISEASE | -1.33 | 0.062 |
| 11 | KEGG_VALINE_LEUCINE_AND_ISOLEUCINE_DEGRADATION | -1.31 | 0.114 |
| 12 | KEGG_TYPE_I_DIABETES_MELLITUS | -1.30 | 0.094 |
| 13 | KEGG_ALZHEIMERS_DISEASE | -1.30 | 0.051 |
| 14 | KEGG_VIRAL_MYOCARDITIS | -1.29 | 0.083 |
| 15 | KEGG_OXIDATIVE_PHOSPHORYLATION | -1.29 | 0.101 |

**Supplementary Table 2. GSEA on homoharringtonine (HHT) treated CD123+.** GSEA analysis of MDS patient samples sorted for Lin-/CD34+/CD38-/CD123+ cells and treated with drug for 6 hours at 200 nM before RNA was isolated and used for RNA-seq studies. Pathway analysis shows decreases in KEGG Lysosome, KEGG Splicesome, KEGG Glutathione Metabolism, and KEGG Oxidative Phosphorylation in the HHT treated samples (A).

| Ref # | MDS Sample | Age/ Sex | Mut | Karotype | IPSS | Blasts |
|---|---|---|---|---|---|---|
| 1 | 314 | 64 M | WT | Complex; FISH del7q and del17p13.1 | 2 (int-2) | 9.5 |
| 2 | 268 | 86 M | SF3B1 SRSF2 | Trisomy 13 | 1.5 (int-2) | 5 |
| 3 | 280 | 62 M | WT | Complex; minus 7, del20q | 3 (high) | 12 |
| 4 | 336 | 77 M | IDH2, ASXL1, U2AF | Gain of 1q21 sequences | NA | 10 |
| 5 | 395 | 78 F | SF3B1 | Chr-7q | NA | 12 |
| 6 | 288 | 66 F | WT for Splice factor, FLT3, and IDH mutations | Chr-5,-7,-8 (complex) | 3 (high) | 11 |
| 7 | 285 | 74 F | SF3B1 | NK | 1 | 0.5 |
| 8 | 298 | 49 F | WT for Splice factor, FLT3, and IDH mutations | t(2;3), -7, | 5.5 | 5 |
| 9 | 148 | 72 M | WT for Splice factor, FLT3, and IDH mutations | NK | 4 | 10 |
| 10 | 250 | 59 F | None detected | Monosomy 7 | 2 (int-2) | 10 |
| 11 | 388 | 82 M | NRAS | Gain of 8 | NA | 5 |
| 12 | 540 | 55 F | SF3B1 | NK | NA | 8 |
| 13 | 501 | 65 M | ASXL1, PTPN11, TET2 | Monosomy 7 | NA | 5 |
| 14 | 833 | 62 F | ASXL1, KDM6A, STAG2, TET2 | NK | NA | 6 |
| 15 | 699 | 80 M | DNMT3A, GATA2, NRAS, RUNX1, U2AF1 | del(7q) | NA | 15 |
| 16 | 628 | 75 F | IDH1, EZH2 | NK | NA | 15 |
| 17 | 520 | 59 F | CEBPA, ASXL1, RUNX1 | Del(9q) | 2.5 | 2 |
| 18 | 905 | 50 M | IDH1,RUNX1 | 47,XY,+8[3]/46,XY[16]. | 6.5 | 9.5 |
| 19 | 878 | 60 M | SRSF2, RUNX1 | NK | NA | 11.5 |
| 20 | 798 | 70 F | None detected | 46,XX,add(5)(q11.2),del(11)(q22q25)[16]/46,XX[4] | NA | 7.5 |
| 21 | 795 | 71 M | EZH2 | NK | NA | 9.0 |
| 22 | 785 | 48 F | DNMT3 | 46,XX,t(6;9)(p23;q34)[20] | NA | 10.5 |
| 23 | 741 | 69 F | ASXL1,TET2 | 46,XX,add(2)(p21),del(4)(q22q28)[7]/46,sl,3~20dmin[3]/46~48,sdl1,+21,2~13dmin[cp2]/ | NA | 6.5 |
| 24 | 061 | 69 M | None detected | 46, XY [20] | NA | NA |

**Supplementary Table 3. Patient Specimen Characteristics**

| Mass | Symbol | DVS Catolog # | Antigen | Clone | Reactivity | Used in SPADE clustering |
|---|---|---|---|---|---|---|
| 142 | Nd | 3142004A | Caspase 3 (Cleaved) | D3E9 | Cross | |
| 143 | Nd | 3143001B | Cd117 | 104D2 | Human | * |
| 144 | Nd | 3144001B | CD11b (Mac-1) | ICRF44 | Human | * |
| 147 | Sm | 3147006B | CD7 | CD7-6B7 | Human | * |
| 148 | Nd | 3148001B | CD34 | 581 | Human | * |
| 150 | Nd | 3150005A | pStat5 [Y694] | 47 | Cross | |
| 151 | Eu | 3151001B | CD123 | 6H6 | Human | * |
| 152 | Sm | 3152005A | pAKT | D9E | Cross | |
| 153 | Eu | 3153008B | TIM-3 | F38-2E2 | Human | * |
| 154 | Sm | 3154001B | CD45 | HI30 | Human | * |
| 156 | Gd | 3156002A | p-p38 [T180/Y182] | D3F9 | Cross | |
| 158 | Gd | 3158001B | CD33 | WM53 | Human | * |
| 159 | Tb | 3159007B | CD90 (Thy-1) | 5E10 | Human | * |
| 163 | Dy | 3163006A | MCL-1 | Polyclonal | Human | |
| 164 | Dy | 3164004A | IkBa | L35A5 | Cross | |
| 166 | Er | 3166006A | pNF-kB p65 [S529] | K10-895.12.50 | | |
| 167 | Er | 3167001B | CD38 | HIT2 | Human | * |
| 168 | Er | 3168001B | Ki-67 | Ki-67 | Human | |
| 169 | Tm | 3169011B | CD19 | HIB19 | Human | * |
| 170 | Er | 3170001B | CD3 | UCHT1 | Human | * |
| 174 | Yb | 3174001B | HLA-DR | L243 | Human | * |
| 176 | Yb | 3176008B | CD56 (NCAM) | NCAM16.2 | Human | * |
| 195 | Pt | | Cell ID Cisplatin | | | |

**Supplementary Table 4. Mass cytometry panel.**
