# Supplementary material for: Characterization and targeting of malignant stem cells in patients with advanced myelodysplastic syndromes
Source: Nat Commun. 2018 Sep 12;9:3694. doi: 10.1038/s41467-018-05984-x (PMC6135858; doi:10.1038/s41467-018-05984-x)
Supplement: Supplementary file 2 — Peer Review File [file 41467_2018_5984_MOESM2_ESM.pdf]

Reviewers' comments:

Reviewer #1 (Remarks to the Author):

Jordan et al demonstrate the importance of CD123+ cells in MDS bone marrow compartment and show that these cells are increased in high risk case; have higher metabolic/protein synthesis rates and correlate with in vivo xenograftability.
There is a dearth of good markers for "malignant" stem cells in MDS and AML and this study advances the field by showing the role of CD123 as an important marker. The studies are praiseworthy due to the difficulties in performing functional and xenografting studies from sorted cells in MDS. The studies are also translational and show novel combinations of bcl2 inh and aza with protein synthesis inhibitors.

Some minor concerns are as follows:

1. Fig 3C: panel with Lin-ve- the gates should be consistent with the other populations

2. In the expression analysis of cd123+ populations, did the authors see any increased expression of STAT3 (will et al, blood), il8 (schinke at al, blood) and il1rap (barreyo et al, Blood); another two candidates that have been seen to be overexpressed in some cases of MDS stem cells. Since MDS is a very heterogenous disease, it is not critical that these will be high, but would be good to know.

3. In the metabolic studies: If the studies were done on lin-/cd34+/cd123+ cells, then the text should reflect that these are stem and progenitors and not just stem (since cd38 not used).

Overall, a very strong and important paper in the field.

Reviewer #2 (Remarks to the Author):

The new study by Drs. Stevens, Jordan and colleagues focused on dissecting the molecular dependencies of malignant hematopoietic stem cells in patients with higher-risk myelodysplastic syndrome (MDS) to uncover novel targets for leukemic stem cell-directed therapy. MDS is not only an adult tissue-specific stem cell-derived disease, it is also currently largely incurable, at least in part due to the insufficient elimination of leukemia-initiating (stem) cells, rendering a better understanding of molecular mechanisms bestowing these leukemic cells with their unique function an immediate clinical need.

The investigators conducted comprehensive molecular and cell physiological analyses into aberrant hematopoietic stem cells in higher-risk MDS (MDS-HSC); the presented experiments are largely well controlled, as well as their resulting data clearly and logically presented; new findings of this study comprise: 1) increased mRNA and cell surface protein expression of CD123 on phenotypical stem cells in higher-risk MDS (similar to their past findings on the aberrant expression of this protein on leukemic stem cells in acute myeloid leukemia (AML)), 2) differential molecular patterning of CD123+ compared to CD123- MDS-HSC, 3) including increased RNA and protein synthesis pathways as well as increased redox-signaling in CD123+ stem cells, 4) without increased cell cycle activation or proliferation, 5) functional reliance of CD123+ MDS-HSCs (but not cord blood-derived healthy HSCs) on increased protein production and redox signaling which could be counteracted therapeutically.

With that, this study provides important new insights into the mechanistic underpinnings of aberrant hematopoietic stem cells in MDS which could have immediate implications for patients with conventional therapy-resistant higher-risk MDS. I have only a few questions and suggestions

for the current manuscript.

Major critique:
1) Figure 1: CD123 overexpression in leukemic HSCs in MDS could be further supported/clarified.
a. What is the overall frequency of patients with higher–risk MDS presenting with CD123+ HSCs?
b. What is the relative frequency of these cells in patients that harbor this population?
c. If possible, assessing another molecular marker (such as an acquired genetic aberration (as done for the in vivo studies presented in Fig. 6) in CD123+ vs. CD123- MDS-HSCs would further buttress the finding that CD123 expression marks aberrant stem cells in higher risk MDS and might allow for a deeper insight into the fraction of genetically abnormal cells overexpressing CD123.

2) Figure 3: Cell cycle analysis may have technical limitations – alternative validation experiment needed to substantiate the finding of unaltered cell cycle activity/proliferation.
a. The measurement of in vivo protein synthesis provided in Fig. 3A is elegant and provides a very interesting insight into the intra- and inter-patient variation of the degree of increased protein synthesis. To better understand how these findings relate to healthy cells, it would be immensely helpful to include OP-PURO incorporation analyses in healthy CD123- HSCs. Is the degree of variation similar this the overall level much decreased? How do protein synthesis rates in normal stem cells compare to more active progenitor cells (Lin-CD34+CD38+)?
b. The frequency of quiescent HSCs measured for and shown in Fig. 3C/D is much above (close to 100%) the previously published ones (80-90%). Given the low-frequency of HSCs in general, I am concerned about the number of events collected for this analysis is too low. How many evets are shown in the density posts of Fig. 3C (lower row).
c. Moreover, to address this surprising observation further, it would be important to measure cell cycle activity by another method. E.g. in live cells by Hoechst 33343 and Pyronin Y incorporation, which based on the data presented here should indicate a higher frequency of CD123+ cells having exited G0 – based on the observed increase RNA synthesis.
d. A snap shot cell cycle analysis such as presented here will also not allow for the assessment of eventual changes occurring in CD123+ MDS-HSCs once they have entered the cell cycle (e.g. time to progress), addition of BrdU/EdU incorporation or live dye dilution assays upon liquid culture would be suitable means to assess whether overall cell cycle activity and proliferation rates are truly unaltered.
e. If these suggested testes are impossible to conduct, the finding needs to be massively tuned down as the data presented thus far do not support the claim well enough.

3) Figure 5/7: Long-term and progenitor effects of OMA/ABT treatment on healthy stem and progenitor cells is unclear.
a. The authors present very impressive data on the inhibitory effects of single agent and combination treatment of MDS-HSC using a protein synthesis inhibitor (OMA) and/or BCL-2 inhibitor (to reduce redox signaling) along with standard of care agent, azacytidine (inclusion of which I found to be a particular plus!) (Fig. A-C).
b. To render the proposed use of protein synthesis inhibitors specific for therapeutic targeting of MDS-HSC – the authors need to provide additional data pertaining to healthy cells that may also rely on protein synthesis (differentiating progenitors). The shown short term effects of the proposed treatment on healthy CD34+ cells (viability upon 24hr liquid culture) are inadequate to assess the effects of protein synthesis inhibition on proliferating normal cells. Please include in vivo colony assay data on the number and differentiation capacity of myeloid progenitors upon treatment, or alternatively more information on multilineage engraftment (experimental data on pan hCD45+ cell engraftment) in Fig. 7C.

4) GEO accession links for the collected transcriptomic and metabolomics analyses are not included in the manuscript. Please add.

Minor critique:
1) Figure 1/Suppl. Fig.1:
a. What are the 14 independent cell surface markers?
b. CD123 is expressed on myeloid progenitor populations (GMP and CMP). It would be more intuitive to show a SPADE analysis of Lin-/CD34+/CD38- cells rather than the used stem and progenitor cell-containing CD34+/Lin- fraction (Fig.1B). It would also be interesting to look at other stem cell markers, such as CD90 and CD49f for differential CD123 expression.
c. Fig. 1D needs an isotype control, or better a healthy control, or lower-risk MDS bone marrow (BM) specimen which allows (as the negative control) for the precise quantification of CD123 expression and positivity if MDS-HSCs. An unstained cell control is not the appropriate control for this type of assessment.

2) Figure 2:
a. As pointed out be the authors, IL1RAP was previously found to be aberrantly expressed in higher-risk MDS and AML HSCs. Have the authors made a similar observation with this gene (or are others of the pathway driving the IL-1 signaling signature)? It would be nice to include this information in the heat map, if possible.
b. Sample #3 in Fig. 2A looks very different in terms of CD123- gene expression profiling compared to #1,2, yet much more similar when compared to gene expression patterns of CD123+ cells (just going by color intensity of the shown #3 CD123+ and #3 CD123- specimen they look even identical in both up and down-regulated genes). Please recheck for technical errors. Alternatively, was the degree of CD123 expression lower in this patient's HSC?
c. Have the authors looked at the overlap of differential gene expression in CD123+ MDS-HSCs with CD123+ AML-HSCs?
d. Color code legend for 2A is missing – please add.

3) Figure 4: Color code legend to Fig. 4C is missing. Please add.
4) Figure 5: panels C and D would greatly benefit from additional information on the actual viable cell numbers upon 24hr culture, and upon a longer liquid culture.

We sincerely thank the reviewers for their many helpful comments and apologize for any oversights or lack of clarity in the original manuscript. We have carefully addressed each point as outlined in the response below (**reviewer comments indicated by bold type**). The manuscript has been revised with the additional of multiple new experiments (4 of the main figures and 7 supplemental figures have been revised to include new data and/or corrections stipulated by reviewers). Revisions to the main manuscript are denoted by red text. Please note, in response to the central issues raised by reviewers, several important new studies have been performed and incorporated as follows:

1. Addressing other known MDS targets: Per reviewer 1 suggestions we have included a more in depth analysis of IL1RAP, STAT3, and IL8 using data from RNA-seq and flow cytometry studies.
2. Additional profiling of drug toxicity in normal cells: We have included additional analysis of normal xenograft toxicity relating to mature cells as well as protein translation rates in normal cells.
3. Cell cycle analysis: We have improved the clarity of the analysis to show a more representative example, as well as an improved graphical representation of the data and individual plots of measured samples.
4. CD123 immunophenotype in MDS: We have done an in depth analysis of mass cytometry data in relation to normal stem and progenitor phenotype and have included the expression of CD123 in MDS patients at varying stages of pathogenesis.

Reviewers' comments:

Reviewer #1 (Remarks to the Author):

Jordan et al demonstrate the importance of CD123+ cells in MDS bone marrow compartment and show that these cells are increased in high risk case; have higher metabolic/protein synthesis rates and correlate with in vivo xenograftability. There is a dearth of good markers for "malignant" stem cells in MDS and AML and this study advances the field by showing the role of CD123 as an important marker. The studies are praiseworthy due to the difficulties in performing functional and xenografting studies from sorted cells in MDS. The studies are also translational and show novel combinations of bcl2 inh and aza with protein synthesis inhibitors.

Some minor concerns are as follows:

1. Fig 3C: panel with Lin-ve- the gates should be consistent with the other populations

We appreciate the reviewer noticing this error. We have corrected the gating. We have also added the cell cycle plots (KI-67 vs DAPI) for seven MDS stem cell specimens in supplemental figure 5. The scale of the combination data in figure 3D has also been adjusted and individual samples are represented in the graph.

2. In the expression analysis of cd123+ populations, did the authors see any increased expression of STAT3 (will et al, blood), il8 (schinke at al, blood) and il1rap (barreyo et al, Blood); another two candidates that have been seen to be overexpressed in some cases of MDS stem cells. Since MDS is a very heterogenous disease, it is not critical that these will be high, but would be good to know.

We appreciate the reviewer's point and have further investigated these 3 pathways:

STAT3: Interestingly and in agreement with the cited studies we see an elevation in STAT3 pathway levels through geneset enrichment analysis. While STAT3 itself is not differentially expressed at the RNA level, the pathway is enriched in CD123+ cells (**supplemental figure 4B**) in agreement with Will et al. We investigate phosphorylated STAT3 (S727) and see a small but significant increase between CD123+ and CD123- cells in 5 MDS patient specimens (**supplemental figure 4C**). Please see modified text in the results section (**top of page 9).**

IL8: IL8 levels via flow cytometry in 5 high risk MDS specimens do not show differential expression between CD123+ and CD123- (**see reviewer Figure 1C below**). We conclude that the elevated IL8 expression previously reported by Schinke et al, is likely a feature of all CD34+ cells in high risk MDS, and not differentially expressed between the CD123+ and CD123- subpopulations.

IL1RAP: As reported by Barreyo at al, IL1RAP is elevated in the -7/7q patient at the RNA level (**see reviewer figure 1A-B below**). The IL1 signaling is also enriched in CD123+ but not due to IL1RAP RNA levels. IL1RAP at the protein level agrees with previous literature in that it is elevated in the CD34+/CD38- subpopulation of the patient with monosomy 7 versus a normal chromosome 7 (see below and **supplemental figure 4D**). However, in this instance we still do not see a differential between CD123+ and CD123- cells.

We also note that elevated STAT5 signaling was observed in the CD123+ cells (**shown in supplemental figure 2C**) and GSEA enrichment shows that STAT signaling is enriched in CD123+(**supplemental table 4B**).

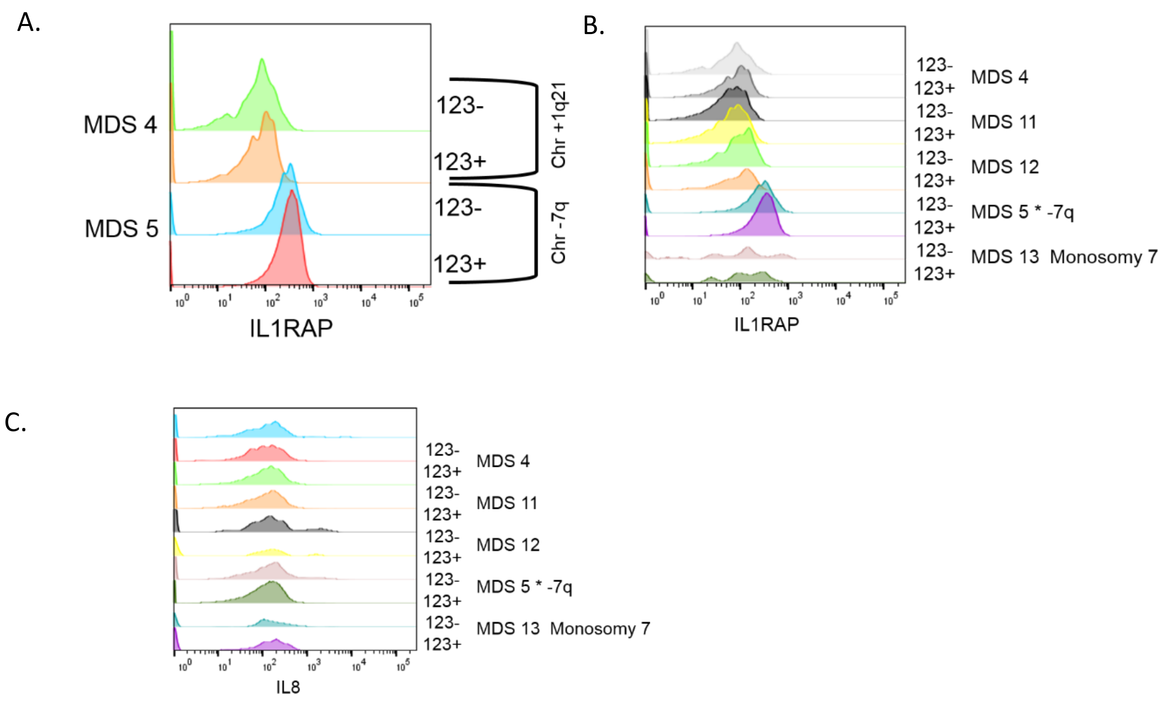

Reviewer figure 1

Flow cytometric analysis of high risk MDS specimens for IL1RAP and IL8. (A) Comparison of a specimen with normal chromosome 7 (MDS 4) and abnormal chromosome 7 (MDS 5) for IL1RAP expression in the Lin-/CD34+/CD38-/CD123+ or Lin-/CD34+/CD38-/CD123- subpopulation. MDS 5 patient shows increased IL1RAP in the primitive cells but no differential between 123+ and 123-. (B) Flow cytometric analysis of additional high risk specimens for IL1RAP, again showing increased IL1RAP in the chromosome 7 abnormal patient and no differential between 123+ and 123-. (C) IL8 staining in 5 high risk MDS specimens showing no differential staining between CD123+ and CD123- subpopulations.

3. In the metabolic studies: If the studies were done on lin-/cd34+/cd123+ cells, then the text should reflect that these are stem and progenitors and not just stem (since cd38 not used).

Thanks for pointing this out, the text has been modified to correct this error.

Reviewer #2 (Remarks to the Author):

The new study by Drs. Stevens, Jordan and colleagues focused on dissecting the molecular dependencies of malignant hematopoietic stem cells in patients with

higher-risk myelodysplastic syndrome (MDS) to uncover novel targets for leukemic stem cell-directed therapy. MDS is not only an adult tissue-specific stem cell-derived disease, it is also currently largely incurable, at least in part due to the insufficient elimination of leukemia-initiating (stem) cells, rendering a better understanding of molecular mechanisms bestowing these leukemic cells with their unique function an immediate clinical need.

The investigators conducted comprehensive molecular and cell physiological analyses into aberrant hematopoietic stem cells in higher-risk MDS (MDS-HSC); the presented experiments are largely well controlled, as well as their resulting data clearly and logically presented; new findings of this study comprise: 1) increased mRNA and cell surface protein expression of CD123 on phenotypical stem cells in higher-risk MDS (similar to their past findings on the aberrant expression of this protein on leukemic stem cells in acute myeloid leukemia (AML)), 2) differential molecular patterning of CD123+ compared to CD123- MDS-HSC, 3) including increased RNA and protein synthesis pathways as well as increased redox-signaling in CD123+ stem cells, 4) without increased cell cycle activation or proliferation, 5) functional reliance of CD123+ MDS-HSCs (but not cord blood-derived healthy HSCs) on increased protein production and redox signaling which could be counteracted therapeutically.

With that, this study provides important new insights into the mechanistic underpinnings of aberrant hematopoietic stem cells in MDS which could have immediate implications for patients with conventional therapy-resistant higher-risk MDS. I have only a few questions and suggestions for the current manuscript.

Major critique:
1) Figure 1: CD123 overexpression in leukemic HSCs in MDS could be further supported/clarified.

a. What is the overall frequency of patients with higher–risk MDS presenting with CD123+ HSCs?

As summarized in the original Figure 1C-D (5 high risk specimens) and the newly added **supplemental figure 1C** (7 additional high risk specimens), we observe that all have at least some up-regulation of CD123 in the CD34+/CD38- subpopulation. In addition, the previous report by Li et al that we cite has extensively documented this finding. In their report, analysis of 41 high risk MDS specimens, showed consistent up-regulation of CD123 in CD34+/CD38- cells[1].

b. What is the relative frequency of these cells in patients that harbor this population?

We have addressed this question by including examples of relative frequency of these populations in 6 samples. This data is in **supplemental figure 1C** and shows that relative frequency is variable amongst total bone marrow, blasts, and the CD34+/CD38- populations. These findings are in agreement with previous findings by Li et al, where analysis of 41 High-risk samples showed a similar frequency (53%) as we describe in supplemental figure 1C.

c. If possible, assessing another molecular marker (such as an acquired genetic aberration (as done for the in vivo studies presented in Fig. 6) in CD123+ vs. CD123- MDS-HSCs would further buttress the finding that CD123 expression marks aberrant stem cells in higher risk MDS and might allow for a deeper insight into the fraction of genetically abnormal cells overexpressing CD123.

We have performed additional studies to address this point. We used flow cytometry to isolate the phenotypically defined CD123+ vs. CD123- stem cell populations from 3 patient specimens. Each sorted population was subjected to cytogenetic analysis to detect the mutation characteristic of the particular specimen. As shown in the table below (**reviewer Table 1**) the data suggest an enrichment in the cytogenetic abnormality in the CD123+ cells in comparison to the CD123- in all three samples. However, only one of the three specimens shows a substantial enrichment, which we appreciate is not terribly compelling. It is of course quite difficult to know which mutations would be most informative at late stages of pathogenesis. As major drivers of disease, events like del 7q and monosomy 5 likely occur early during disease development (i.e. prior to progression to high risk disease) and therefore would not necessarily reveal changes between CD123+ and CD123- subpopulations in high risk specimens. While other mutations might be more informative, they are likely more specific to individual patients. We are not aware of any broadly prevalent mutational events that would serve to delineate changes within high risk stage patients. Therefore, while we appreciate this important suggestion by the reviewer, we respectfully suggest that technical challenges in performing this type of work are beyond the scope of the present study.

Patient Specimen	Abnormality	Lin-/CD34+/CD38-/CD123-	Lin-/CD34+/CD38-/CD123+	Bulk
MDS 314	Deletion 7q	95.0 %	99.0 %	76.5 %
MDS 288	Monosomy 5	20.1 %	58.5 %	0.5 %
MDS 280	Minus 7	95.0 %	99.5 %	29.0 %

Reviewer table 1: Cytogenetics of sorted patient specimens. Patient bone marrow specimens sorted for specified populations were subjected to cytogenetic analysis via FISH for a particular genetic abnormality. Percent represents amount of abnormality of each immunophenotype.

2) Figure 3: Cell cycle analysis may have technical limitations – alternative validation experiment needed to substantiate the finding of unaltered cell cycle activity/proliferation.

a. The measurement of in vivo protein synthesis provided in Fig. 3A is elegant and provides a very interesting insight into the intra- and inter-patient variation of the degree of increased protein synthesis. To better understand how these findings relate to healthy cells, it would be immensely helpful to include OP-PURO incorporation analyses in healthy CD123- HSCs. Is the degree of variation similar this the overall level much decreased? How do protein synthesis rates in normal stem cells compare to more active progenitor cells (Lin-CD34+CD38+)?

We appreciate this insightful comment and have performed additional studies to address these points. We now include a more in depth analysis of patient sample translation rates and in vivo (xenograft) translation rates (see **supplemental figure 9B-C**), as well as normal bone marrow translation rates (**supplemental figure 10B)**. Interestingly, we consistently see that CD123+ MDS stem cells have increased translation over other populations present including lymphocytes. It appears that monocytes have significantly more translation than MDS stem cells (**supplemental figure 9B**). In normal bone marrow, as previously reported by Signer et al[2] for mouse hematopoiesis, the most primitive cells (CD34+/CD38-) have the lowest rates of translation (supplemental figure 10B), a striking contrast to MDS. Further, analysis of protein translation rates in xenografts (**suppl figure 9C**) show similarly elevated rates in MDS cells as observed for freshly isolated specimens (**Figure 3A-B and suppl 9B**). We have revised the text in the results section to further comment on these data.

b. The frequency of quiescent HSCs measured for and shown in Fig. 3C/D is much above (close to 100%) the previously published ones (80-90%). Given the low-frequency of HSCs in general, I am concerned about the number of events collected for this analysis is too low. How many evets are shown in the density posts of Fig. 3C (lower row).

We apologize that our data was not presented more clearly. The reviewer is absolutely correct, the cycle activity shown in our original figure was unusually low. We were focused on the comparative analysis (i.e. showing that CD123+ vs. CD123- subpopulations did not differ) and neglected to carefully quantify our data (the use of contour plots artificially reduced low frequency events). The figure has been corrected to include higher resolution analysis and gating, which now indicates the expected percentages of cells in varying stages of cycle (**Figures 3C**). In addition, we have added the averages for each cycle stage shown in **Figure 3D** to more clearly present this data. We appreciate the reviewer catching this problem.

To further document cell cycle status, we have also included the KI67 vs DAPI plots for all of specimens presented in Figure 3D (see **supplemental figure 5**). As shown in **Figure 3D and S5**, the percentage of cells in G0 is variable and ranges from 80-95%. The data also shows that when combined there are not significant differences between CD123+ and CD123- rates.

c. Moreover, to address this surprising observation further, it would be important to measure cell cycle activity by another method. E.g. in live cells by Hoechst 33343 and Pyronin Y incorporation, which based on the data presented here should indicate a higher frequency of CD123+ cells having exited G0 – based on the observed increase RNA synthesis.

We attempted to use HO/PY labeling, but unfortunately the resolution of this technique was considerably lower than the Ki67/DAPI labeling shown in **figures 3C, 3D, and S5**. With the improved presentation noted above for **figures 3C, 3D, and S5**, we hope the reviewer will find our conclusions valid. Please note, the main point we wish to convey is that there is no discernable difference in cell cycle activity between the CD123+ vs. CD123- stem cell populations.

d. A snap shot cell cycle analysis such as presented here will also not allow for the assessment of eventual changes occurring in CD123+ MDS-HSCs once they have entered the cell cycle (e.g. time to progress), addition of BrdU/EdU incorporation or live dye dilution assays upon liquid culture would be suitable means to assess whether overall cell cycle activity and proliferation rates are truly unaltered.

We appreciate this interesting point. We employed EdU staining on MDS specimens, but in agreement with the static analysis (**Figure 3C-D**) do not see marked differences in cycle rates. This point has been added to the results section.

e. If these suggested tests are impossible to conduct, the finding needs to be massively tuned down as the data presented thus far do not support the claim well enough.

With the additional data and corrections noted above, we believe it is valid to conclude that there is no significant difference in cycle rate between the CD123+ and CD123- stem cell populations. The text has been carefully reviewed and edited to ensure that our conclusions are appropriately stated.

3) Figure 5/7: Long-term and progenitor effects of OMA/ABT treatment on healthy stem and progenitor cells is unclear.
a. The authors present very impressive data on the inhibitory effects of single agent and combination treatment of MDS-HSC using a protein synthesis inhibitor (OMA) and/or BCL-2 inhibitor (to reduce redox signaling) along with standard of care agent, azacytidine (inclusion of which I found to be a particular plus!) (Fig. A-C).
b. To render the proposed use of protein synthesis inhibitors specific for therapeutic targeting of MDS-HSC – the authors need to provide additional data pertaining to healthy cells that may also rely on protein synthesis (differentiating progenitors). The shown short term effects of the proposed treatment on healthy CD34+ cells (viability upon 24hr liquid culture) are inadequate to assess the effects of protein synthesis inhibition on proliferating normal cells. Please include in vivo colony assay data on the number and differentiation capacity of myeloid progenitors upon treatment, or alternatively more information on multilineage engraftment (experimental data on pan hCD45+ cell engraftment) in Fig. 7C.

We thank the reviewer for this suggestion and agree that in order for this regimen to be clinically viable the normal cell toxicities need to be limited. We have performed additional analyses of cells obtained from the xenograft studies and observe that the toxicities found in figure 7 (decreases in hCD45+ cells) are largely due to a decrease in CD19+ cells (now shown in newly added **suppl Figure 10A**). This finding is evident for both the omacetaxine + ABT treatment and the omacetaxine + azacitidine treatment. There is no loss of myeloid cells (CD33+), and perhaps even a slight gain. The overall toxicities to normal cells are far less than the effects seen to MDS stem cells. Also, as shown in **Figure 7**, there is no significant loss of normal HSCs with either regimen.

Interestingly, investigation of translation rates in normal bone marrow reveals increased rates in lineage + cells and low rates in normal HSCs (**supplemental figure 10**). These results are consistent with the minor toxicities for normal HSCs found in this study.

Lastly, we note that all the agents used in our study are approved for various leukemias and have shown favorable toxicity profiles in humans. The use of oma+ aza in AML has been explored in humans with preliminary results (limited patient numbers) showing promising efficacy and limited toxicity. Taken together, we believe the data indicate that these regimens will be sufficiently tolerable to permit clinical use.

4) GEO accession links for the collected transcriptomic and metabolomics analyses are not included in the manuscript. Please add.

All transcriptomic data has been submitted to the GEO database (accession number: GSE116100).

Minor critique:
1) Figure 1/Suppl. Fig.1:
a. What are the 14 independent cell surface markers?

We have noted the 14 surface markers with a star in **supplemental table 4** in a new column.

b. CD123 is expressed on myeloid progenitor populations (GMP and CMP). It would be more intuitive to show a SPADE analysis of Lin-/CD34+/CD38- cells rather than the used stem and progenitor cell-containing CD34+/Lin- fraction (Fig.1B). It would also be interesting to look at other stem cell markers, such as CD90 and CD49f for differential CD123 expression.

We have included a new spade tree in **supplemental figure 2A** from independent patient samples. This tree shows the makeup of the CD34+ population and is colored for CD123 expression and notes the traditional progenitor and stem cell populations in new clusters. We did not have CD49f available for use in our panel but do have CD90 and the CD90 cluster is noted in the figure.

c. Fig. 1D needs an isotype control, or better a healthy control, or lower-risk MDS bone marrow (BM) specimen which allows (as the negative control) for the precise quantification of CD123 expression and positivity if MDS-HSCs. An unstained cell control is not the appropriate control for this type of assessment.

We have addressed this point with a new **figure 1D**. We have added an isotype control and fluorescence minus one control to the figure showing CD123 staining in MDS stem cells.

2) Figure 2:
a. As pointed out be the authors, IL1RAP was previously found to be aberrantly expressed in higher-risk MDS and AML HSCs. Have the authors made a similar observation with this gene (or are others of the pathway driving the IL-1 signaling signature)? It would be nice to include this information in the heat map, if possible.

We have added the respective heatmaps for the IFN alpha, beta, and gamma gene sets and have also added the IL1 signaling gene set in **supplemental figure 4**. The geneset enrichment we see in CD123+ cells is not due to differential expression of L1RAP at the RNA or protein level. Interestingly, IL1RAP appears to be up in the both CD123+ and CD123- in the deletion 7q patient (3) and less so in the other -7 patient (1). This partially fits with the previous finding showing IL1RAP in chromosome 7 modified patients, but without differential expression in CD123+ cells. This could be due to numerous reasons, but interestingly the signaling as suggested by geneset enrichment analysis is up in CD123+ subpopulation. We also see increased IL1RAP in a monosomy 7 patient via flow cytometry in the CD34+/CD38- subpopulation but no differential in expression between CD123+ and CD123- (**supplemental figure 4D**).

b. Sample #3 in Fig. 2A looks very different in terms of CD123- gene expression profiling compared to #1,2, yet much more similar when compared to gene expression patterns of CD123+ cells (just going by color intensity of the shown #3 CD123+ and #3 CD123- specimen they look even identical in both up and down-regulated genes). Please recheck for technical errors. Alternatively, was the degree of CD123 expression lower in this patient's HSC?

We appreciate the reviewer's thorough review and this indeed was a technical error. We have corrected the heatmap (**figure 2A**) reflecting non-duplicate values and have adjusted the text to reflect the difference in enriched genes. We have checked the other pieces of transcriptomic data and this was the only data with duplicate values.

c. Have the authors looked at the overlap of differential gene expression in CD123+ MDS-HSCs with CD123+ AML-HSCs?

Interestingly, we see some overlap with AML and CD123+ cells. As shown in **supplemental table 1**, a signature for AML is enriched in CD123+ cells. We do not have specific data relating to transcriptomics for CD123+ AML LSCs but using available gene sets we see an enrichment in CD123+ MDS cells for the GENTLES_LEUKEMIA_STEMCELL_UP signature (see below).

Gene set	Size	ES	NES	NOM p-val	FDR q-val
GENTLES_LEUKEMIC_STEM_CELL_UP	27	0.50	1.49	0.043	0.363

d. Color code legend for 2A is missing – please add.

We have added the scale bar to the figure.

3) Figure 4: Color code legend to Fig. 4C is missing. Please add.

We have added the scale bar to the figure.

4) Figure 5: panels C and D would greatly benefit from additional information on the actual viable cell numbers upon 24hr culture, and upon a longer liquid culture.

We have included example culture conditions and viability in **supplemental figure 3**. This figure notes the overnight culture viability to be approximately 70-80% overall. It also shows the distribution of cells in the respective gates upon culture. These conditions have been optimized for myeloid growth through inclusion of cytokines in serum free conditions. This example is consistent amongst the samples used for viability studies.

Citations

1. Li LJ*, et al.* Increased CD34+ CD38− CD123+ cells in myelodysplastic syndrome displaying malignant features similar to those in aml. *International journal of hematology* **100**, 60-69 (2014).

2. Signer RA, Magee JA, Salic A, Morrison SJ. Haematopoietic stem cells require a highly regulated protein synthesis rate. *Nature* **509**, 49 (2014).

REVIEWERS' COMMENTS:

Reviewer #1 (Remarks to the Author):

Revision addresses all points satisfactorily

Reviewer #2 (Remarks to the Author):

I would like to thank the authors for the very careful revision of their manuscript. The investigators have addressed the critique raised previously.

The only request I am left with at this point is the addition of the data obtained from the conducted EdU experiments (pertaining to Figure 3).

Again, we sincerely thank the reviewers for their excellent comments. Below, we have addressed the final remaining point for reviewer #2.

REVIEWERS' COMMENTS:

Reviewer #1 (Remarks to the Author):

Revision addresses all points satisfactorily

Reviewer #2 (Remarks to the Author):

I would like to thank the authors for the very careful revision of their manuscript. The investigators have addressed the critique raised previously.

The only request I am left with at this point is the addition of the data obtained from the conducted EdU experiments (pertaining to Figure 3).

The requested EdU data has been added to the manuscript (supplemental Figure 5B).
